# Early antiplatelet therapy after intravenous thrombolysis for acute ischemic stroke: a systematic review and meta-analysis

**DOI:** 10.1007/s10072-024-07821-0

**Published:** 2024-10-29

**Authors:** Hesham Kelani, Ahmed Naeem, Rowan H. Elhalag, Mohamed Abuelazm, Nadia Albaramony, Ahmed Abdelazeem, Mohammad El-Ghanem, Travis R. Quinoa, Diana Greene-Chandos, Ketevan Berekashvili, Ambooj Tiwari, Arthur D. Kay, David P. Lerner, Lisa R. Merlin, Fawaz Al-Mufti

**Affiliations:** 1 Neurology Department, SUNY DOWNSTATE Health Science University, One Brooklyn Health, Brooklyn, NY USA; 2 Al-Azhar Faculty of Medicine, Asyut, Egypt; 3 Alexandria Faculty of Medicine, Alexandria, Egypt; 4 Faculty of Medicine, Tanta University, Tanta, Egypt; 5 Neurology and Neurocritical Care Department, Mayo Clinic, Jacksonville, FL USA; 6 Department of Neurology, Sanford USD Medical Center, Sanford, SD USA; 7 University of Houston, HCA Houston-Northwest Medical Center, Houston, TX USA; 8 Department of Neurosurgery, Rutgers New Jersey School of Medicine, Newark, NJ USA; 9 Department of Neurology, School of Medicine, University of Saint Louis, Saint Louis, MO USA; 10 Department of Neurology, NYU Grossman School of Medicine, New York, NY USA; 11 Departments of Neurology and Physiology and Pharmacology, SUNY Downstate Health Sciences University, Brooklyn, NY USA; 12 Neurosurgery Department, Westchester Medical Center, Westchester, New York, NY USA

**Keywords:** Early antiplatelet therapy, Alteplase, Ischemic stroke, Safety, Effectiveness, Meta-analysis

## Abstract

**Background:**

Early neurological deterioration (END) and recurrence of vessel blockage frequently complicate intravenous thrombolysis (IVT) for acute ischemic stroke (AIS). Several studies have indicated the potential effectiveness of the early initiation (within < 24 h) of antiplatelet therapy (APT) after IVT. However, conflicting results have been reported by other studies. We aimed to offer a thorough overview of the current literature through a systematic review and meta-analysis.

**Methods:**

Our systematic review and meta-analysis were prospectively registered on PROSPERO (ID: CRD42023488173) following the PRISMA guidelines. We systematically searched Web of Science, SCOPUS, PubMed, and Cochrane Library until May 5, 2024. Rayyan. ai facilitated the screening process. The R statistical programming language was used to calculate the odds ratios and conduct a meta-analysis. Our primary outcomes were excellent functional recovery (modified Rankin Scale score 0–1), symptomatic intracranial hemorrhage (sICH), and mortality.

**Results:**

Eight studies involving 2,134 participants were included in the meta-analysis. Early APT showed statistically significant increased odds of excellent functional recovery (mRS 0–1) compared to the standard APT group (OR, 1.81; [95% CI: 1.10, 2.98], *p* = 0.02). However, we found no differences between the early and standard APT groups regarding sICH (OR, 1.74; [95% CI: 0.91, 3.33], *p* = 0.10) and mortality (OR, 0.88; [95% CI: 0.62, 1.24]; *p* = 0.47).

**Conclusion:**

Early APT within 24 h of IVT in stroke patients is safe, with no increase in bleeding risk, and has a positive effect on excellent functional recovery. However, there was a statistically insignificant trend of increased sICH with early APT, and the current evidence is based on highly heterogeneous studies. Further large-scale RCTs are warranted.

**Supplementary Information:**

The online version contains supplementary material available at 10.1007/s10072-024-07821-0.

## Introduction

Stroke is the second leading cause of mortality worldwide. In 2019, 3.29 million deaths were attributed to ischemic stroke, and is anticipated to rise to 4.90 million by the year 2030 [1].

Intravenous thrombolysis (IVT) with alteplase (rtPA) improves the functional outcome and decreases mortality and morbidity. Thus, it remains the primary treatment modality for acute ischemic stroke (AIS) within 4.5 h, a recommendation endorsed by both the American Heart Association (AHA) and the European Stroke Organization (ESO) [2–5].

Despite the significance of IVT in stroke care, the initial 24 h following the administration of intravenous recombinant tissue-type plasminogen activator (tPA) exhibit a diverse clinical course, with a majority of patients showing improvement, some maintaining a stable condition, and approximately 10% undergoing early neurological deterioration (END) [6]. Besides, revascularization rates following its administration are estimated to be less than 50%. Moreover, the risk of arterial re-occlusion looms, with potential rates reaching as high as 60% [7–12]. This instant or delayed re-occlusion is linked to endothelial injuries and platelet activation, leading to in situ thrombo-occlusion [13].

The current literature presents conflicting findings regarding the early initiation of antiplatelet therapy (APT) after IVT concerning the risk of cerebral hemorrhage and functional outcomes. This systematic review and meta-analysis aimed to explore the safety and efficacy of early (< 24h) APT in patients with AIS after IVT or bridging therapy.

## Methodology

### Study protocol

Our review process was registered and documented on PROSPERO under the ID (CRD42023488173). We conducted a thorough systematic review and meta-analysis, adhering to the guidelines provided by the Preferred Reporting Items for Systematic Reviews and Meta-Analyses (PRISMA) statement [14]. The PRISMA 2020 checklist is presented in **(**Table S1**)**.

### Literature search

Web of Science, SCOPUS, PubMed (MEDLINE), and Cochrane Central Register of Controlled Trials (CENTRAL) were systematically searched from their inception until May 6, 2024. The following keywords and their MeSH terms were used to formulate the search strategy: “stroke”, “thrombolysis”, “antiplatelet”, and “early administration”. The detailed search approach is presented in (Table S2**)**.

### Study selection and eligibility criteria

The results of the four database searches were imported into Rayyan.ai and duplicates were automatically detected and removed. Title and abstract screening was performed using Rayyan.ai to determine the remaining results and their relevance to the meta-analysis. Relevant studies were exported to a Microsoft Excel sheet, and their full-text papers were obtained and entered the full-text screening phase according to the inclusion criteria for the final eligibility for qualitative and quantitative analysis [15]. Two reviewers independently conducted all screening phases, and the first author resolved conflicts.

We included randomized controlled trials (RCTs) and observational studies, published in English, comparing early APT (within 24 h) with standard initiation (> 24 h) after rt-PA IVT in AIS, either alone or for bridging endovascular thrombectomy. Studies were excluded if they were secondary research articles (systematic reviews and editorials), studied endovascular thrombectomy without rt-PA IVT, assessed the early initiation of anticoagulants(e.g., heparin), or did not report data on any of our primary outcomes. Primary outcomes of interest were excellent functional recovery (modified Rankin Scale score of ≤ 1, mRS 0–1), symptomatic intracranial hemorrhage (sICH), any intracranial hemorrhage (ICH), and mortality at 90 days. No restrictions were imposed regarding the selection of APT within the drug family. Our focus was primarily centered on investigating the timing of initiation of APT.

### Data extraction

We used a prespecified Excel sheet to extract the following data from each included study: author’s name, study design, number of participants, age of participants, sex of participants, rt-PA type and dosage, type of anti-platelet and dosage, baseline NIHSS, primary outcomes, previous stroke, pre-stroke APT use, onset to IVT, hypertension, diabetes mellitus, dyslipidemia, coronary artery disease, and current smoking.

### Risk of bias assessment

Two reviewers assessed the methodological quality of the included studies. Randomized controlled trials were evaluated using Cochrane’s Risk-of-bias tool (RoB 2.0), while the Risk of Bias in Non-randomized Studies of Interventions tool (ROBINS-I) was used to assess the quality of the included observational studies [16, 17].

### Statistical analysis

Using the R statistical programming language, we calculated the odds ratios (ORs) with the corresponding 95% confidence interval (CI), and a meta-analysis with the Mantel–Haenszel method was performed for each outcome [18]. A *p*-value of < 0.05 was identified for statistical significance. Heterogeneity was assessed using the tau squared and I^2^ statistic, with I^2^ ≥ 60% indicating a substantial heterogeneity. We used the Leave-One-Out test and subgroup analyses to assess the robustness of our findings.

## Results

### Search results and screening

**Fig. 1:**
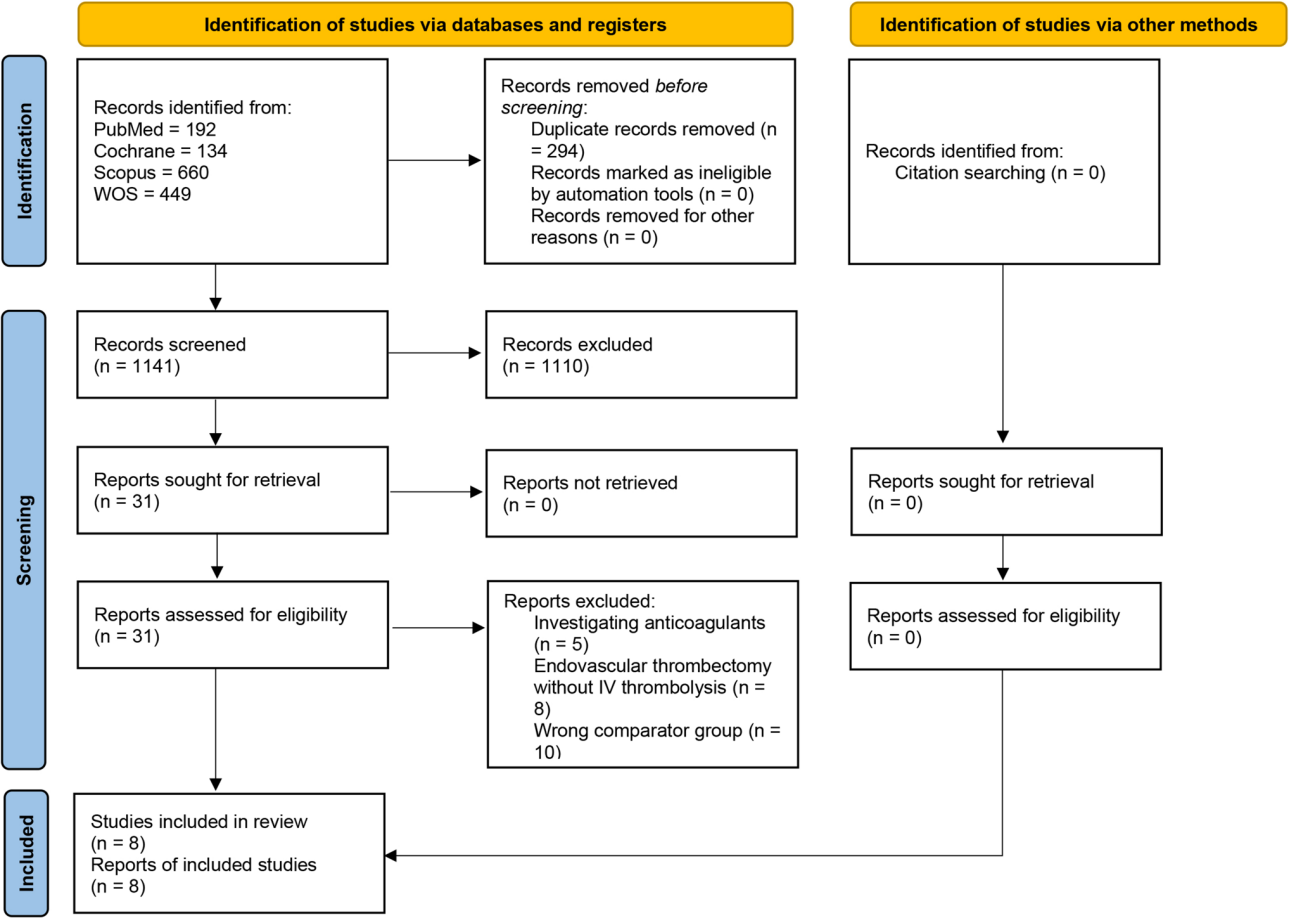
PRISMA flow chart of the screening process

The four database searches yielded 1,435 records. After we removed 294 duplicates, 1,141 were screened for the title and abstract. One thousand one hundred ten records were found irrelevant, and the full texts of 31 studies were retrieved. By the end of the full-text screening phase, 23 studies did not fulfill our inclusion criteria, and eight were included in the meta-analysis (Fig. 1). A summary of the included studies is presented in (Table 1).

**Table 1:** Summary of included studies

Study ID	Study design	Country	Total participants, N	Intravenous thrombolysis		Early APT group		Standard APT group		Baseline NIHSS, mean ± SD		Primary Outcome(s)	sICH definition	QA & ROB
				Agent/Dose	Time window	Antiplatelet agent/Dose	Administration time	Antiplatelet agent/Dose	Administration time	Early APT group	Standard APT group			
Zhang et al. 2022 [20]	RCT	China	73	Alteplase, 0.9 mg/kg	Within 4.5 h of stroke onset	Tirofiban, a loading dose of 0.4 µg/kg/minute over 30 min followed by a maintenance dose of 0.1 µg/kg/minute up to 24 h	Immediately after END was diagnosed within 24 h after thrombolysis	Aspirin at a dose of 100 mg plus clopidogrel at a dose of 75 mg per day	24 h after intravenous thrombolysis	8.90 ± 2.75	8.14 ± 3.51	mRS at 90 days, sICH, and any ICH	NR	High
Liu et al. 2019 [19]	RCT	China	240	Alteplase, 0.9 mg/kg	Within 4.5 h of stroke onset	Tirofiban, 5 mg/kg administered as an intravenous bolus dose followed by an intravenous infusion of 0.1 µg/kg/min for 24 h	Within 24 h after thrombolysis	No antiplatelet therapy within the first 24 h after thrombolysis	NR	9.48 ± 4.62	10.38 ± 4.68	mRS at 90 days, sICH, and any ICH	NR	High
Pancioli et al. 2013 [23]	RCT	USA	126	Alteplase, (0.6 mg/kg for early APT group, 0.9 mg/kg foe standard APT group)	NR	Eptifibatide (135 mcg/kg bolus followed by a 2-h infusion at 0.75 mcg/kg per minute)	Coadministered with alteplase	No antiplatelet therapy within the first 24 h after thrombolysis	NR	16.6 ± 8.64	13.6 ± 8.27	sICH, any ICH, and mortality	Any hemorrhage detected on CT or MRI that is associated with clinical deterioration with an increase of ≥ 1 point on NIHSS or any hemorrhage leading to death within 36 h of treatment	Some concern
Zinkstok et al. 2012	RCT	Netherlands	642	Alteplase, 0.9 mg/kg	Within 4.5 h of stroke onset	Aspirin, 300 mg intravenously	Within 90 min after alteplase	Oral aspirin	24 h after intravenous thrombolysis	9.6 ± 7.4	9.3 ± 6.7	mRS at 90 days, sICH, and serious systemic bleeding	Neurological deterioration of 4 points or more increase on the NIHSS in combination with intracranial hemorrhage on follow-up CT scan	Low
Amaro et al. 2013	Observational study	Spain	77	Alteplase	Within 4.5 h of stroke onset	Oral aspirin (100 mg/day), oral clopidogrel (75 mg/day, preceded by a loading dose of 300 mg)	Within 24 h after thrombolysis	Oral aspirin (100 mg/day), oral clopidogrel (75 mg/day, preceded by a loading dose of 300 mg)	24 h after intravenous thrombolysis	7.66 ± 8.38	8.00 ± 6.28	sICH and any ICH	Neurological deterioration of 4 points or more increase on the NIHSS in combination with intracranial hemorrhage on follow-up CT scan	Moderate
Wells et al. 2022 [21]	Observational study	USA	300	Alteplase	NR	Aspirin, clopidogrel, ticagrelor, or aspirin/dipyridamole	Within 24 h after thrombolysis	Aspirin, clopidogrel, ticagrelor, or aspirin/dipyridamole	24 h after intravenous thrombolysis	11.33 ± 20.30	12.00 ± 23.14	sICH	Any blood on the CT or MRI scan combined with a clinical deterioration and increase of ≥ 4 points in the NIHSS score or leading to death	Serious
Chuanjie Wu et al. 2019	Observational study	China	187	Alteplase, 0.9 mg/kg	Within 4.5 h of stroke onset	Tirofiban (5 mg diluted with 100 mL of normal saline) was usually administered intravenously with a bolus of 0.25 to 0.5 mg (5–10 mL) at a rate of 1 mL/minute, and then followed by a continuous infusion of 0.25 to 0.5 mg/hour	Within 24 h after thrombolysis	No antiplatelet therapy within the first 24 h after thrombolysis	NR	7.00 ± 5.25	7.00 ± 4.54	mRS at 90 days, sICH, and any ICH	Neurological deterioration of 4 points or more increase on the NIHSS in combination with intracranial hemorrhage on follow-up CT scan	Serious
Krastev et al. 2024 [24]	Observational study	Slovakia	489	Alteplase, 0.9 mg/kg	NR	Oral aspirin (100 mg/day) and/or clopidogrel (75 mg/day), which could be preceded by a loading dose of 300 mg	Within 24 h after thrombolysis	Oral aspirin (100 mg/day) and/or clopidogrel (75 mg/day), which could be preceded by a loading dose of 300 mg	24 h after intravenous thrombolysis	7.95 ± 4.82	9.35 ± 5.33	sICH, mortality, and mRS scores at 90 days	Any blood on the CT or MRI scan combined with clinical deterioration and increase of ≥ 4 points in the NIHSS score or leading to death, within 36 h of treatment	Low

*APT* Antiplatelet therapy, *NIHSS* National Institute of Health Stroke Scale, *QA* Quality assessment, *ROB* Risk of bias, *mRS* modified Rankin Scale, *END* Early neurological deterioration, *SD* Standard deviation, *sICH* symptomatic intracranial hemorrhage, *NR* Not reported, *CT* Computed Tomography, *MRI* Magnetic Resonance Imaging

### Characteristics of included studies

The eight studies (four RCTs and four observational studies) included 2,134 participants, of which 1,339 (62.7%) participants were assigned to the early APT group and 795 (37.3%) were assigned to the standard APT group. All studies used alteplase as the IVT agent. Regarding the APT agent, three studies used tirofiban, three used aspirin plus clopidogrel, one used aspirin alone, and one used eptifibatide. The mean age of participants ranged from 61.4 to 72.46 years. Male sex represents 53.8% of the total participants. The mean baseline NIHSS ranged (from 7 to 16.6). Baseline characteristics of the included studies are shown in (Table 2).

**Table 2:** Baseline characteristics of the included studies

Study ID	Groups	Number of participants, n	Age, years, (mean ± SD)	Sex, n		Previous Stroke, n	Prestroke antiplatelet use, n	Baseline NIHSS, (mean ± SD)	IVT plus thrombectomy, n	HTN, n	DM, n	Dyslipidemia, n	CAD, n	Current Smoking, n	SBP, mm.Hg, (mean ± SD)	DBP, mm.Hg, (mean ± SD)	Onset to IVT, min, (mean ± SD)	TOAST classification
				M	F													
Zhang et al. 2022 [20]	Early APT	59	69.24 ± 14.88	29	30	7	14	8.90 ± 2.75	NR	45	10	14	16	28	161.20 ± 15.51	92.58 ± 12.69	196.10 ± 53.89	Large artery atherosclerosis = 36 Small vessel occlusion = 24
	Standard APT	14	68.21 ± 12.86	7	7	4	2	8.14 ± 3.51	NR	11	4	3	0	3	158.57 ± 24.25	93.71 ± 26.23	209.00 ± 55.17	Large artery atherosclerosis = 12 Small vessel occlusion = 2
Amaro et al. 2013	Early APT	52	68.33 ± 11.98	32	20	5	20	7.66 ± 8.38	10	34	13	22	8	15	155 ± 22.87	NR	134.33 ± 68.61	Cardioembolism = 18 Atherothrombotic = 10 Lacunar = 6 Undetermined = 12 Other = 4
	Standard APT	25	71.66 ± 11.62	15	10	3	12	8.00 ± 6.28	4	19	6	9	3	5	153.66 ± 30.65	NR	136.66 ± 62.88	Cardioembolism = 8 Atherothrombotic = 4 Lacunar = 3 Undetermined = 8 Other = 1
Wells et al. 2022 [21]	Early APT	100	62.1 ± 14.3	46	54	24	35	11.33 ± 20.30	26	78	36	NR	NR	30	NR	NR	NR	NR
	Standard APT	200	61.4 ± 14	96	104	66	66	12.00 ± 23.14	8	154	61	NR	NR	55	NR	NR	NR	NR
Chuanjie Wu et al. 2019	Early APT	121	63.8 ± 13.2	83	38	NR	14	7.00 ± 5.25	NR	85	33	40	12	28	153.9 ± 18.8	84.4 ± 14.2	165.1 ± 34.6	Large artery atherosclerosis = 33 Small vessel occlusion = 50 Cardioembolism = 17
	Standard APT	66	61.5 ± 12.6	43	23	NR	15	7.00 ± 4.54	NR	37	23	23	13	21	150.8 ± 20.3	79.5 ± 13.3	157.7 ± 38.7	Large artery atherosclerosis = 23 Small vessel occlusion = 21 Cardioembolism = 10
Liu et al. 2019 [19]	Early APT	177	67.41 ± 7.07	97	1	22	57	9.48 ± 4.62	NR	129	49	55	12	44	NR	NR	NR	Large artery atherosclerosis = 114 Small vessel occlusion = 30 Cardioembolism = 24
	Standard APT	63	67.71 ± 6.72	35	0	10	23	10.38 ± 4.68	NR	45	21	15	5	11	NR	NR	NR	Large artery atherosclerosis = 45 Small vessel occlusion = 10 Cardioembolism = 6
Pancioli et al. 2013 [23]	Early APT	101	70.4 ± 17.59	53	48	14	43	16.6 ± 8.64	NR	84	31	NR	12	31	155 ± 30.08	84.33 ± 21.81	NR	NR
	Standard APT	25	72.46 ± 16.42	13	12	2	7	13.6 ± 8.27	NR	19	9	NR	4	7	146 ± 18.08	82.33 ± 12.57	NR	NR
Zinkstok et al. 2012	Early APT	322	67.1 ± 13.8	163	159	11	NR	9.6 ± 7.4	NR	140	38	36	NR	NR	155.6 ± 22.4	83.4 ± 13.1	124.3 ± 54.4	NR
	Standard APT	320	66.7 ± 13.5	160	160	4	NR	9.3 ± 6.7	NR	129	26	35	NR	NR	156.5 ± 21.6	48.5 ± 14.00	127.9 ± 57.6	NR
Krastev et al. 2024 [24]	Early APT	407	69.81 ± 13.15	233	174	NR	NR	7.95 ± 4.82	NR	313	131	NR	NR	NR	158.07 ± 27.19	85.10 ± 14.29	NR	NR
	Standard APT	82	70.56 ± 10.47	45	37	NR	NR	9.35 ± 5.33	NR	73	31	NR	NR	NR	164.42 ± 28.43	89.78 ± 17.36	NR	NR

*NIHSS* National Institute of Health Stroke Scale, *IVT* Intravenous thrombolysis, *HTN* Hypertension, *DM* Diabetes mellitus, *CAD* Coronary artery disease, *SBP* Systolic blood pressure, *DBP* Diastolic blood pressure, *TOAST* Trial of ORG 10172 in acute stroke statement, *SD* Standard deviation, *NR* Not reported

### Risk of bias assessment

**Fig. 2:**
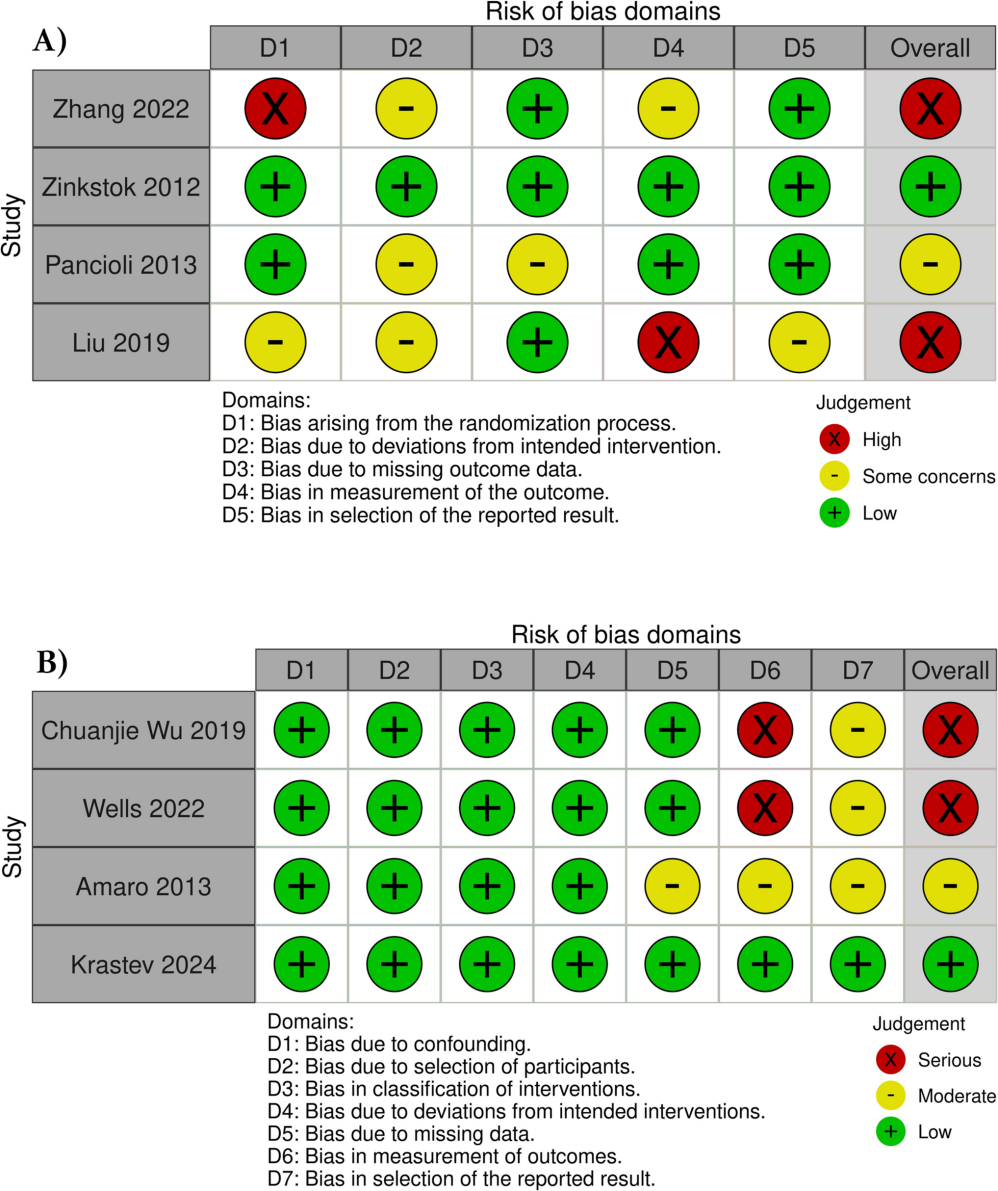
Assessment of risk of bias in the included studies. **A** ROB-2 evaluation of RCTs. The panel presents a schematic representation of risks (low = green, unclear = yellow, and high = red) for specific types of biases of each study in the review. **B** ROBINS-1 evaluation of observational studies. The panel presents a schematic representation of risks (low = green, moderate = yellow, and serious = red) for specific types of biases of each study in the review

The overall risk of bias was high in four studies [19–22], low in two study [23, 24], and moderate in two studies [25, 26]. The ROB details for all the included studies are shown in (Fig. 2).

### Safety outcomes

#### Symptomatic ICH

**Fig. 3:**
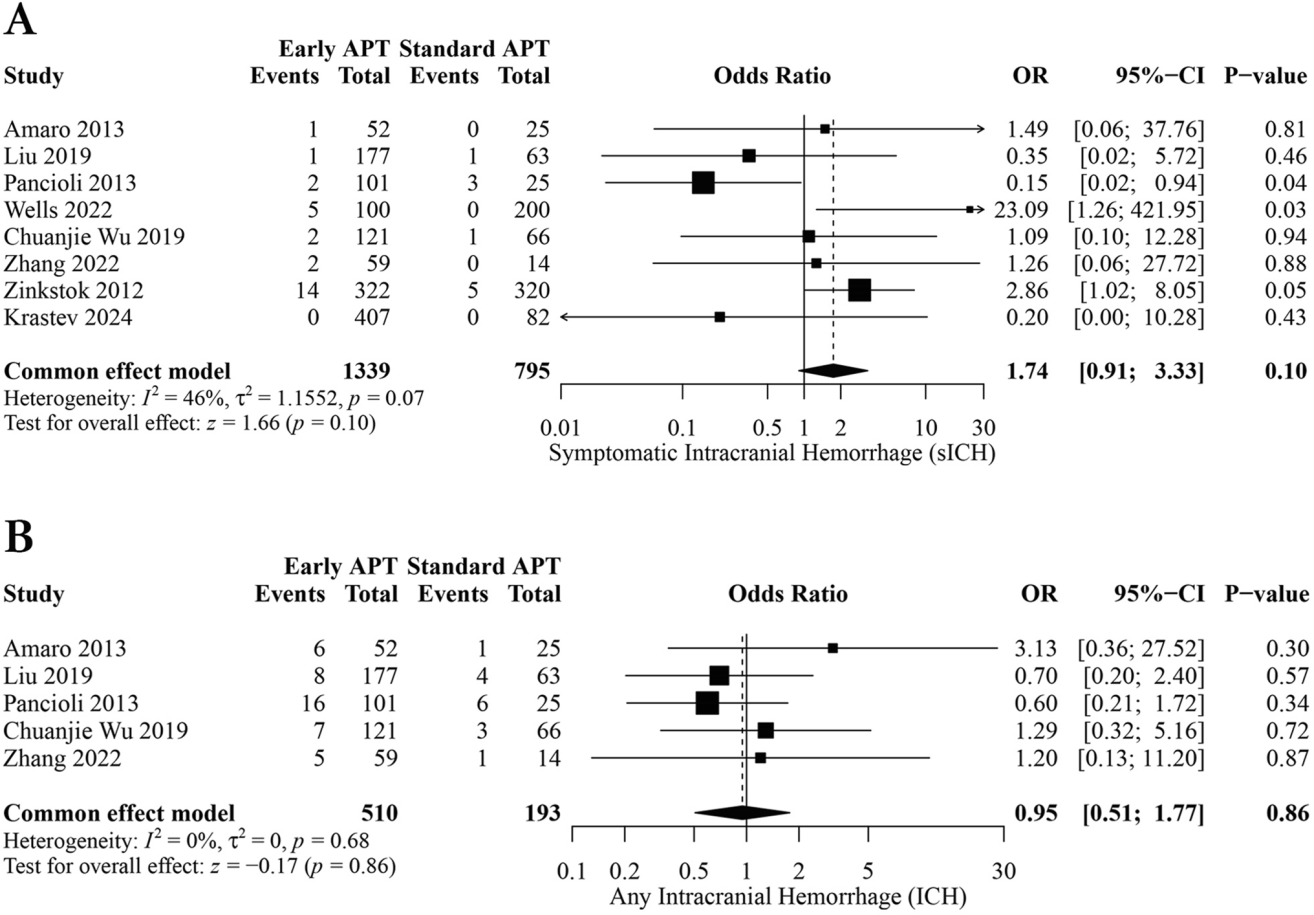
Forest plots of meta-analyses for: **A** Symptomatic intracranial hemorrhage (sICH). **B** Any intracranial hemorrhage (ICH)

Eight studies involving 2,134 patients reported sICH. No statistically significant difference was found between the early and standard APT groups (OR, 1.74; [95% CI: 0.91, 3.33], *p* = 0.10). The pooled studies were homogeneous (I^2^ = 46%; *P* = 0.07) (Fig. 3A).

Remarkably, upon the exclusion of (Pancioli et al., 2013) [23], a statistically significant increase in sICH emerged (OR, 2.48; [95% CI: 1.18, 5.20], *p* = 0.02). Simultaneously, the heterogeneity index was reduced to I^2^ = 5%. These findings imply that the inclusion of (Pancioli et al., 2013) [23] played a significant role in the observed heterogeneity, and its omission led to a more homogeneous and statistically significant association between early APT and sICH occurrence (Fig. S1).

#### Any ICH

Five studies involving 703 patients reported any ICH incidence. We found no statistically significant difference between the early and standard APT groups (OR, 0.95; [95% CI: 0.51, 1.77]; *p* = 0.86) (Fig. 3B). The pooled studies were homogeneous (I^2^ = 0%; *p* = 0.68). The sensitivity analysis proved the robustness of this finding (Fig. S2).

#### Other systemic bleeding

Five studies involving 1,442 patients reported other systemic bleeding events. No statistically significant difference was found between the early and standard APT groups (OR, 0.75; [95% CI: 0.33, 1.71]; *p* = 0.50) (Fig. S3). The pooled studies were homogeneous (I^2^ = 0%, *p* = 0.98). The sensitivity analysis proved the robustness of this finding (Fig. S4).

#### Mortality

**Fig. 4:**
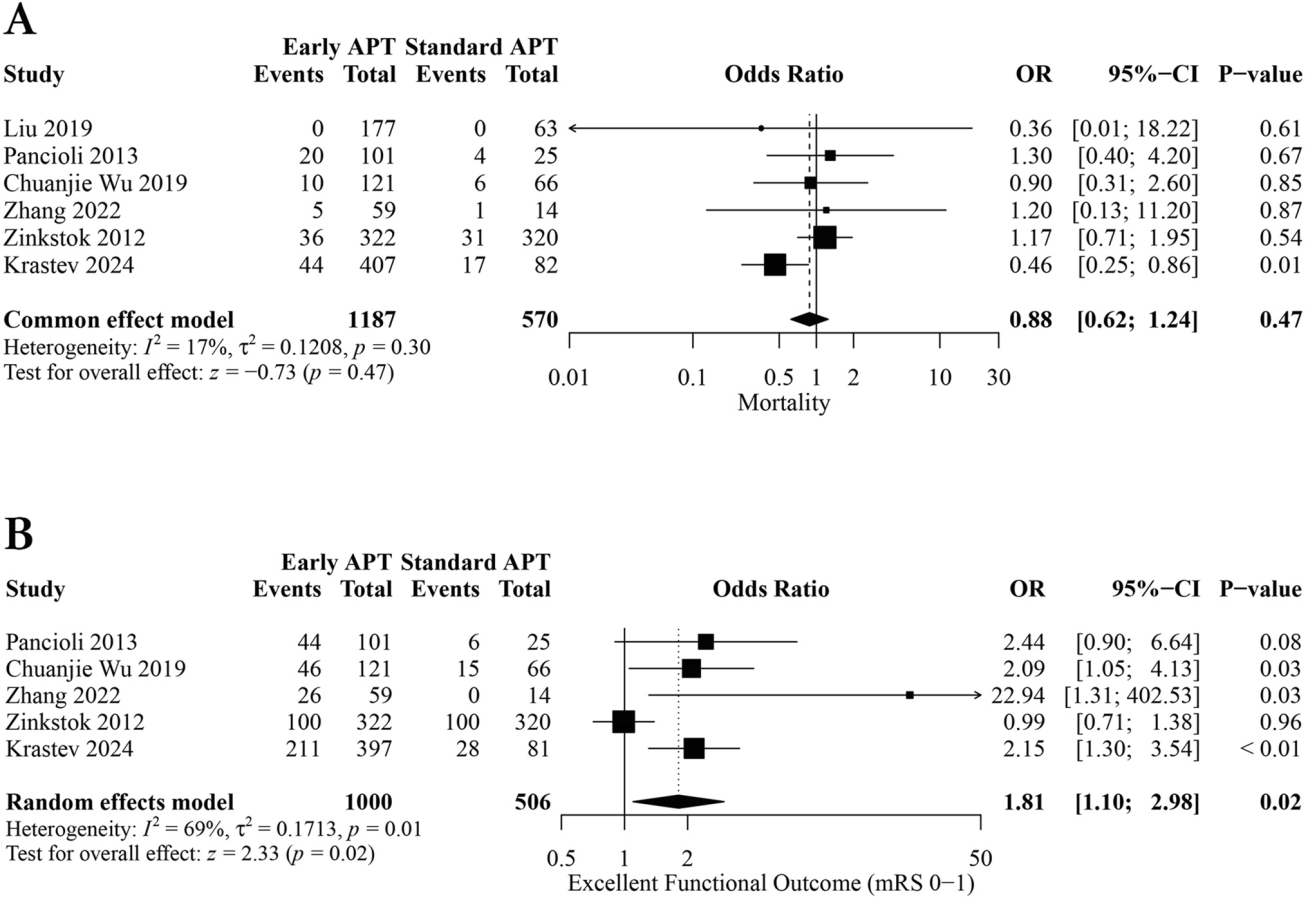
of Forest plots of meta-analyses for: **A** Mortality at 90 days. **B** Excellent functional recovery (mRS 0–1)

Six studies involving 1,757 patients reported mortality at 90 days. No statistically significant difference was found between the early and standard APT groups (OR, 0.88; [95% CI: 0.62, 1.24]; *p* = 0.47) (Fig. 4A). The pooled studies were homogeneous (I^2^ = 17%; *p* = 0.30). Sensitivity analysis proved the robustness of this finding (Fig. S5).

### Efficacy outcomes

#### Excellent functional recovery

Five studies involving 1,506 patients reported excellent functional recovery using a modified Rankin Scale score (mRS) of 0–1 at 90 days. Early APT showed statistically significant increased odds of excellent functional recovery compared to the standard APT group (OR, 1.81; [95% CI: 1.10, 2.98], *p* = 0.02) (Fig. 4B). The pooled studies were heterogeneous (I^2^ = 69%; *P* = 0.01).

This finding wasn’t robust through sensitivity analysis. Notably, after the omission of (Zinkstok et al., 2012), the heterogeneity index (I^2^) was decreased to 0% (OR, 2.25; [95% CI: 1.56, 3.27], *p* < 0.01), suggesting that the observed heterogeneity was, in part, attributable to the inclusion of (Zinkstok et al., 2012) (Fig. S6).

#### Good functional recovery

Five studies involving 1,620 patients reported good functional recovery using a modified Rankin Score (mRS) of 0–2 at 90 days. No statistically significant difference was found between the early and standard APT groups (OR, 1.68; [95% CI: 0.97, 2.91], *p* = 0.07) (Fig. S7). The pooled studies were heterogeneous (I^2^ = 79%; *P* < 0.01). Notably, after omitting the study by (Zinkstok et al., 2012), the association between early APT and a good functional outcome became statistically significant (OR, 2.00; [95% CI: 1.23, 3.27]; *p* < 0.01). However, heterogeneity wasn’t resolved (I^2^ = 65%) (Fig. S8).

#### Poor functional recovery

Five studies involving 1,733 patients reported poor functional recovery using a modified Rankin Score (mRS) of 3–6 at 90 days. We found no statistically significant difference between the early and standard APT groups (OR, 0.72; [95% CI: 0.27, 1.93]; *p* = 0.52) (Fig. S9). The pooled studies were heterogeneous (I^2^ = 86%, *P* < 0.01). Sensitivity analysis proved the robustness of this finding, however, the observed heterogeneity persisted without resolution (Fig. S10).

### Subgroup analyses

Subgroup analyses were performed based on study design and mean baseline NIHSS scores for all outcomes. The test for subgroup difference was significant only in mortality (*p* = 0.04) according to the study design. Subgroup analyses for all outcomes are shown in (Figs. S11-S23). Regarding the subgroup analysis of sICH based on study design, observational studies revealed a non-significant trend towards an increased risk of sICH (OR, 3.20; [95% CI: 0.87, 11.80]; *p* = 0.08) with moderate heterogeneity (I^2^ = 30%). Conversely, RCTs exhibited a more modest risk for sICH (OR, 1.36; [95% CI: 0.63, 20.91]; *p* = 0.44), alongside substantial heterogeneity (I^2^ = 64%). These results suggest that while observational studies suggest a potential association, RCTs do not provide sufficient evidence to support a definitive relationship between early APT and sICH risk (Fig. S11).

## Discussion

IVT is the mainstay in stroke management and is recommended by the current guidelines. However, it is restricted by a strict time window, low reperfusion rates, and END [24]. END incidence was reported up to 10%, with 73% mainly derived from recanalized vessel re-occlusion [27, 28]. Therefore, early APT following IVT seems plausible to prevent this re-occlusion and END [27]. Nevertheless, early APT can also increase the risk of hemorrhagic transformation [27].

Our meta-analysis of eight studies, including 2,134 patients, showed that early APT within 24 h following IVT increased the odds of excellent neurological recovery compared to delayed APT. However, there was no difference between both regimens regarding good functional recovery (mRS 0–2), poor functional recovery (mRS 3–6), any ICH, symptomatic ICH, systematic bleeding, and mortality. However, it is important to note the observed trend towards an increase in symptomatic ICH in the early APT group. Although the results did not reach statistical significance, the trend suggests a potential risk that warrants further investigation. Statistical significance may be absent due to the limited sample size and study variability.

Regarding neurological recovery, our analysis was mainly weighted by (Zinkstok et al., 2012) (the ARTIS trial) [26]. Also, the ARTIS trial can be the main reason behind the heterogeneity of the pooled results. To clarify, after excluding the ARTIS trial from the analysis, using leave-one-out sensitivity analysis, the pooled results showed that an early APT regimen enhances good functional recovery, with resolved heterogeneity. This can be explained by the fact that the ARTIS trial investigated intravenous aspirin, while the other studies mainly investigated glycoprotein (GP) IIb/IIIa antagonists (eptifibatide and tirofiban) [20, 21, 23].

Furthermore, the ARTIS trial administered intravenous aspirin at the ultra-early stage (67 ± 25 min after the start of alteplase treatment) [26]. With a half-life of less than five minutes and complete elimination within 60 min, alteplase is rapidly cleared from the plasma [29]. Therefore, the risk of hemorrhage fades within hours, and ultra-early administration of APT as with ARTIS can increase the risk of negative outcomes [22]. The ARTIS, therefore, was prematurely terminated due to the increased risk of symptomatic ICH [26]. Contradictory to aspirin, blocking inhibiting cyclooxygenase-1 (COX1), blocking thromboxane A2 production and thereby, the subsequent hemostatic cascade, GP IIb/IIIa inhibitors specifically inhibit the platelet aggregation by blocking fibrinogen-platelet crosslinking [20]. Thus, GP IIb/IIIa inhibitors have a better hemostatic safety profile [30]. Also, the effect of GP IIb/IIIa inhibitors on platelet function can recover within seven hours from withdrawal [22]. This and our results support the efficacy of GP IIb/IIIa inhibitors instead of aspirin as an early APT following IVT.

Regarding safety outcomes, early APT following IVT was safe with similar rates of ICH, systematic bleeding, and mortality. Of the included studies, only the ARTIS trial and (Wells et al., 2022) [21] showed an increased incidence of sICH with early APT [21, 26]. In the ARTIS, this was mainly derived from the intervention regimen as previously clarified. At the same time, in Wells et al., [21]  this effect was only noticed in patients who received endovascular thrombectomy with four out of five cases of sICH undergoing endovascular thrombectomy [21]. However, after excluding (Pancioli et al., 2013) [23], early APT increased the risk of sICH. The study by (Pancioli et al., 2013) [23] was an underpowered phase 2 trial without balanced recruitment and they mentioned that the rates of sICH were unexpected [23]. Our results come in line with a previous meta-analysis of three trials, with 1,008 patients by (Liu et al.) [31], which found no difference between early and delayed APT regimens in various outcomes [31]. However, a noteworthy departure from the previous study’s findings is our identification of a statistically significant increase in excellent functional recovery associated with early APT.

## Limitations

Our analysis is limited by the following: first, we include non-randomized observational studies liable to various types of bias; however, we conducted subgroup analysis based on the study design between randomized and non-randomized studies. Second, the early APT regimens varied significantly which can explain the heterogeneity in some outcomes. Third, stroke severity throughout the included studies had a wide range of NIHSS; however, we conducted subgroup analysis based on mean baseline NIHSS score. Fourth, due to the lack of data, we could not control some confounding variables, such as infarction site, occluded blood vessel, and APT start timing. Fifth, all included studies used alteplase as an IVT, with no data regarding tenecteplase (TNK). Finally, the role of early anti-platelet after endovascular thrombectomy, especially in patients with large vessel occlusion, remains unknown.

## Implications for future research

Several knowledge gaps remain to be addressed: first, the optimal early APT regimen is still vague; with the current evidence dependent on highly heterogeneous studies. The optimal APT drug is still a matter of debate with the current American Heart Association (AHA)/ American Stroke Association (ASA) guidelines recommending the early infusion of double APT (aspirin plus clopidogrel) within 12–24 h of symptom onset and at least within seven days of onset in patients with recent minor stroke (NIHSS score ≤ 3) or high-risk TIA (ABCD2 score ≥ 4) for 21 to 90 days followed by single APT [28]. Therefore, randomized trials comparing different early APT regimes are still warranted. Second, the timing of the early infusion, especially after the onset of END is still unclear [22]. An unexplained END is currently reported to be associated with susceptibility vessel sign extension on MRI after IVT [32]. Hence, further trials including END patients are required to clarify the optimal early APT timing [22]. Third, the efficacy and safety of early APT regimens after IVT using TNK are still unknown, warranting further research especially when TNK was reported to be more effective than alteplase [5, 33]. Finally, the efficacy and safety of early APT regimens after endovascular thrombectomy also require further investigation.

Some of the previous questions may be answered with the ongoing Early Antiplatelet for minor Stroke following Thrombolysis (EAST) trial [27]. EAST will include patients with minor stroke (NIHSS < 5) and investigate DAPT given after six hours from IVT [27].

## Conclusion

Early APT within 24 h of IVT in stroke patients is safe with no increase in bleeding risk; besides, it has a positive effect on excellent functional recovery. However, there was a statistically insignificant trend of increased sICH with early APT, and the current evidence is based on highly heterogeneous studies with limited data on commonly used antiplatelets (ASA or ASA + clopidogrel). This warrants further large-scale randomized controlled trials investigating the optimal APT regimen, infusion timing, and efficacy after TNK after endovascular thrombectomy.

## Supplementary Information

Below is the link to the electronic supplementary material.

Supplementary file1 (PDF 2568 KB)
