# Supplementary material for: Early antiplatelet therapy after intravenous thrombolysis for acute ischemic stroke: a systematic review and meta-analysis
Source: Neurol Sci. 2024 Oct 29;46(2):617–31. doi: 10.1007/s10072-024-07821-0 (PMC11772446; doi:10.1007/s10072-024-07821-0)
Supplement: Supplementary file 1 — Supplementary file1 (PDF 2568 KB) [file 10072_2024_7821_MOESM1_ESM.pdf]

Supplementary file

Content

Table S1: PRISMA statement.
Table S2: Databases search approach.
Figure S1: Forest plot of sensitivity analysis for sICH.
Figure S2: Forest plot for sensitivity analysis of any ICH.
Figure S3: Forest plot for meta-analysis of other systemic bleedings outcome.
Figure S4: Forest plot for sensitivity analysis of other systemic bleedings outcome.
Figure S5: Forest plot for sensitivity analysis of mortality at 90 days.
Figure S6: Forest plot for sensitivity analysis of excellent functional outcome (mRS 0-1).
Figure S7: Forest plot for meta-analysis of good functional outcome (mRS 0-2).
Figure S8: Forest plot for sensitivity analysis of good functional outcome (mRS 0-2).
Figure S9: Forest plot for meta-analysis of poor functional outcome (mRS 3-6).
Figure S10: Forest plot for sensitivity analysis of poor functional outcome (mRS 3-6).
Figure S11: Subgroup analysis based on study design for sICH.
Figure S12: Subgroup analysis based on baseline NIHSS for sICH.
Figure S13: Subgroup analysis based on study design for any ICH.
Figure S14: Subgroup analysis based on baseline NIHSS for any ICH.
Figure S15: Subgroup analysis based on study design for other systemic bleedings.
Figure S16: Subgroup analysis based on baseline NIHSS for other systemic bleedings outcome.
Figure S17: Subgroup analysis based on study design for mortality.
Figure S18: Subgroup analysis based on baseline NIHSS for mortality.
Figure S19: Subgroup analysis based on study design for mRS 0-1 outcome.
Figure S20: Subgroup analysis based on baseline NIHSS for mRS 0-1 outcome.
Figure S21: Subgroup analysis based on study design for mRS 0-2 outcome.
Figure S22: Subgroup analysis based on study design for mRS 3-6 outcome.
Figure S23: Subgroup analysis based on baseline NIHSS for mRS 3-6 outcome.

Section and Topic	Item #	Checklist item	Location where item is reported
**TITLE**			
Title	1	Identify the report as a systematic review.	Page 1
**ABSTRACT**			
Abstract	2	See the PRISMA 2020 for Abstracts checklist.	Page 2
**INTRODUCTION**			
Rationale	3	Describe the rationale for the review in the context of existing knowledge.	Page 3
Objectives	4	Provide an explicit statement of the objective(s) or question(s) the review addresses.	Page 3
**METHODS**			
Eligibility criteria	5	Specify the inclusion and exclusion criteria for the review and how studies were grouped for the syntheses.	Page 3, 4
Information sources	6	Specify all databases, registers, websites, organisations, reference lists and other sources searched or consulted to identify studies. Specify the date when each source was last searched or consulted.	Page 3
Search strategy	7	Present the full search strategies for all databases, registers and websites, including any filters and limits used.	Supplementary table S2
Selection process	8	Specify the methods used to decide whether a study met the inclusion criteria of the review, including how many reviewers screened each record and each report retrieved, whether they worked independently, and if applicable, details of automation tools used in the process.	Page 3, 4
Data collection process	9	Specify the methods used to collect data from reports, including how many reviewers collected data from each report, whether they worked independently, any processes for obtaining or confirming data from study investigators, and if applicable, details of automation tools used in the process.	Page 4
Data items	10a	List and define all outcomes for which data were sought. Specify whether all results that were compatible with each outcome domain in each study were sought (e.g. for all measures, time points, analyses), and if not, the methods used to decide which results to collect.	Page 4 .
	10b	List and define all other variables for which data were sought (e.g. participant and intervention characteristics, funding sources). Describe any assumptions made about any missing or unclear information.	Page 4
Study risk of bias assessment	11	Specify the methods used to assess risk of bias in the included studies, including details of the tool(s) used, how many reviewers assessed each study and whether they worked independently, and if applicable, details of automation tools used in the process.	Page 4
Effect measures	12	Specify for each outcome the effect measure(s) (e.g. risk ratio, mean difference) used in the synthesis or presentation of results.	Page 4
Synthesis methods	13a	Describe the processes used to decide which studies were eligible for each synthesis (e.g. tabulating the study intervention characteristics and comparing against the planned groups for each synthesis (item #5)).	Page 4
	13b	Describe any methods required to prepare the data for presentation or synthesis, such as handling of missing summary statistics, or data conversions.	Page 4
	13c	Describe any methods used to tabulate or visually display results of individual studies and syntheses.	Page 5, 6
	13d	Describe any methods used to synthesize results and provide a rationale for the choice(s). If meta-analysis was performed, describe the model(s), method(s) to identify the presence and extent of statistical heterogeneity, and software package(s) used.	Page 5,6
	13e	Describe any methods used to explore possible causes of heterogeneity among study results (e.g. subgroup analysis, meta-regression).	Page 5,6

Section and Topic	Item #	Checklist item	Location where item is reported
	13f	Describe any sensitivity analyses conducted to assess robustness of the synthesized results.	Page 5
Reporting bias assessment	14	Describe any methods used to assess risk of bias due to missing results in a synthesis (arising from reporting biases).	Page 5
Certainty assessment	15	Describe any methods used to assess certainty (or confidence) in the body of evidence for an outcome.	Not applicable
**RESULTS**			
Study selection	16a	Describe the results of the search and selection process, from the number of records identified in the search to the number of studies included in the review, ideally using a flow diagram.	Page 4, 5
	16b	Cite studies that might appear to meet the inclusion criteria, but which were excluded, and explain why they were excluded.	Not applicable
Study characteristics	17	Cite each included study and present its characteristics.	Page 5
Risk of bias in studies	18	Present assessments of risk of bias for each included study.	Page 5
Results of individual studies	19	For all outcomes, present, for each study: (a) summary statistics for each group (where appropriate) and (b) an effect estimate and its precision (e.g. confidence/credible interval), ideally using structured tables or plots.	Page 5,6,7,8
Results of syntheses	20a	For each synthesis, briefly summarise the characteristics and risk of bias among contributing studies.	Pages 6
	20b	Present results of all statistical syntheses conducted. If meta-analysis was done, present for each the summary estimate and its precision (e.g. confidence/credible interval) and measures of statistical heterogeneity. If comparing groups, describe the direction of the effect.	Pages 4,5,6
	20c	Present results of all investigations of possible causes of heterogeneity among study results.	Pages 4,5,6
	20d	Present results of all sensitivity analyses conducted to assess the robustness of the synthesized results.	4,5,6
Reporting biases	21	Present assessments of risk of bias due to missing results (arising from reporting biases) for each synthesis assessed.	Page 5,6
Certainty of evidence	22	Present assessments of certainty (or confidence) in the body of evidence for each outcome assessed.	Not applicable
**DISCUSSION**			
Discussion	23a	Provide a general interpretation of the results in the context of other evidence.	Page 7,8
	23b	Discuss any limitations of the evidence included in the review.	Page 7,8
	23c	Discuss any limitations of the review processes used.	Page 7,8
	23d	Discuss implications of the results for practice, policy, and future research.	Page 8
**OTHER INFORMATION**			
Registration and protocol	24a	Provide registration information for the review, including register name and registration number, or state that the review was not registered.	Page 3
	24b	Indicate where the review protocol can be accessed, or state that a protocol was not prepared.	Page 3
	24c	Describe and explain any amendments to information provided at registration or in the protocol.	Not applicable
Support	25	Describe sources of financial or non-financial support for the review, and the role of the funders or sponsors in the review.	Page 8,9

Section and Topic	Item #	Checklist item	Location where item is reported
Competing interests	26	Declare any competing interests of review authors.	Page 8
Availability of data, code and other materials	27	Report which of the following are publicly available and where they can be found: template data collection forms; data extracted from included studies; data used for all analyses; analytic code; any other materials used in the review.	Page 8

Table S1: PRISMA statement 2020.

Author	Database	Search strategy	Results	Date
Ahmed Naeem	Pubmed	(stroke OR "ischemic stroke" OR "brain Ischemic" OR "brain infarction" OR "cerebral ischemia" OR "cerebral infarction") AND (alteplase OR tpa OR "tissue plasminogen activator" OR "IV thrombolysis" OR "intravenous thrombolysis" OR "intra-venous thrombolysis") AND (antiplatelet OR anti-platelet OR "platelet inhibitors" OR DAPT OR "dual antiplatelet" OR dual-antiplatelet OR aspirin OR ASA OR clopidogrel OR ticagrelor OR prasugrel) AND (early OR timing OR intimation OR "early use" OR "early initiation")	192	May 6, 2024
Ahmed Naeem	Scopus	(stroke OR "ischemic stroke" OR "brain Ischemic" OR "brain infarction" OR "cerebral ischemia" OR "cerebral infarction") AND (alteplase OR tpa OR "tissue plasminogen activator" OR "IV thrombolysis" OR "intravenous thrombolysis" OR "intra-venous thrombolysis") AND (antiplatelet OR anti-platelet OR "platelet inhibitors" OR DAPT OR "dual antiplatelet" OR dual-antiplatelet OR aspirin OR ASA OR clopidogrel OR ticagrelor OR prasugrel) AND (early OR timing OR intimation OR "early use" OR "early initiation")	660	May 6, 2024
Ahmed Naeem	WOS	(stroke OR "ischemic stroke" OR "brain Ischemic" OR "brain infarction" OR "cerebral ischemia" OR "cerebral infarction") AND (alteplase OR tpa OR "tissue plasminogen activator" OR "IV thrombolysis" OR "intravenous thrombolysis" OR "intra-venous thrombolysis") AND (antiplatelet OR anti-platelet OR "platelet inhibitors" OR DAPT OR "dual antiplatelet" OR dual-antiplatelet OR aspirin OR ASA OR clopidogrel OR ticagrelor OR prasugrel) AND (early OR timing OR intimation OR "early use" OR "early initiation")	449	May 6, 2024
Ahmed Naeem	Cochrane	(stroke OR "ischemic stroke" OR "brain Ischemic" OR "brain infarction" OR "cerebral ischemia" OR "cerebral infarction") AND (alteplase OR tpa OR "tissue plasminogen activator" OR "IV thrombolysis" OR "intravenous thrombolysis" OR "intra-venous thrombolysis") AND (antiplatelet OR anti-platelet OR "platelet inhibitors" OR DAPT OR "dual antiplatelet" OR dual-antiplatelet OR aspirin OR ASA OR clopidogrel OR ticagrelor OR prasugrel) AND (early	134	May 6, 2024

		OR timing OR intimation OR "early use" OR "early initiation")		

Table(S2): Search strategy, results, and dates.

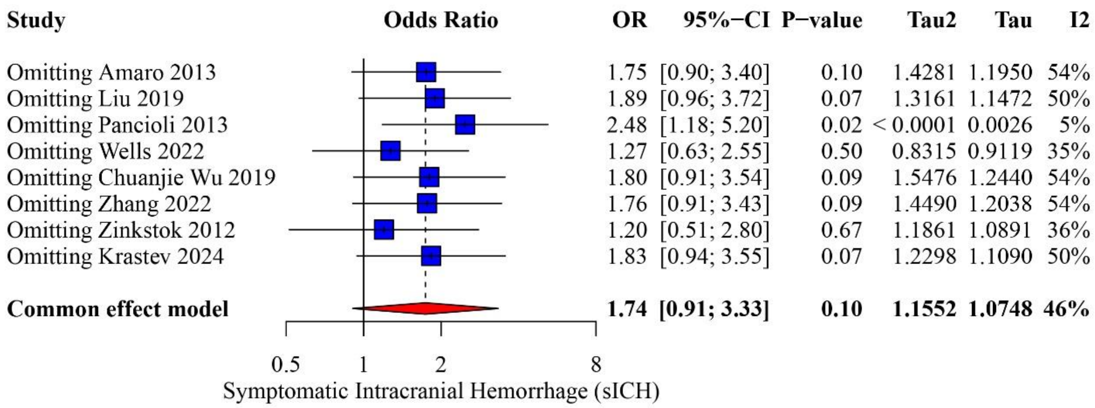

Figure S1: Forest plot of sensitivity analysis for sICH.

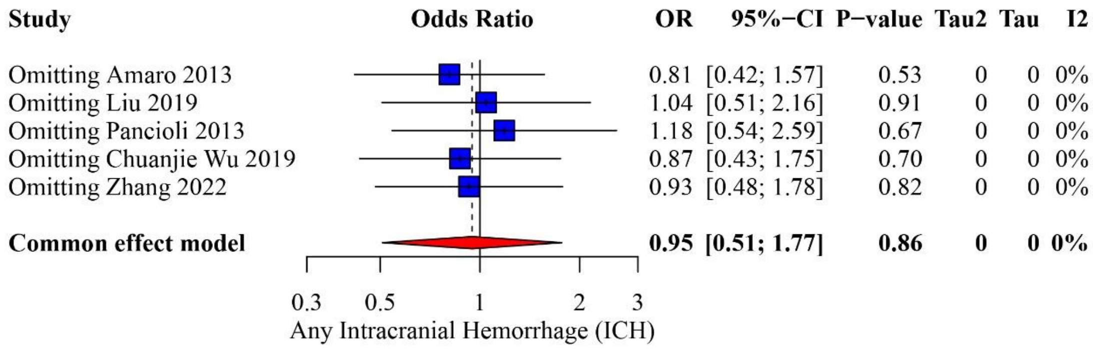

Figure S2: Forest plot for sensitivity analysis of any ICH

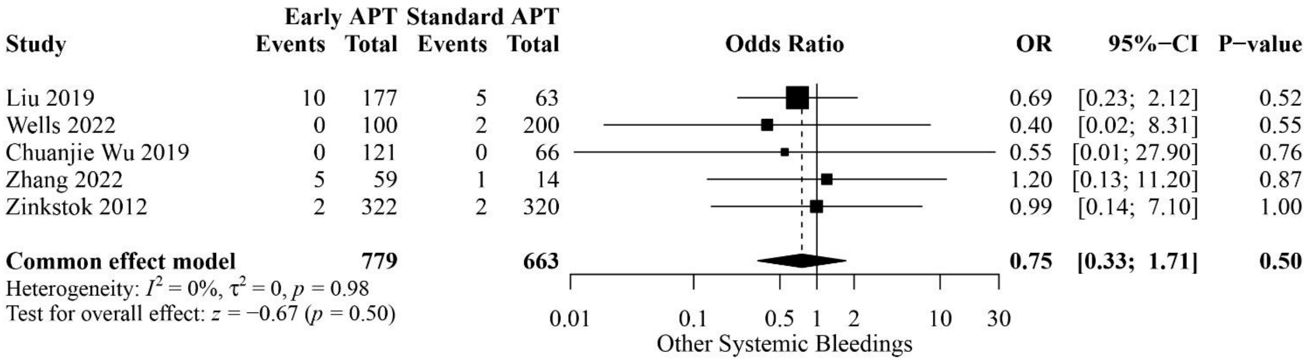

Figure S3: Forest plot for meta-analysis of other systemic bleedings outcome.

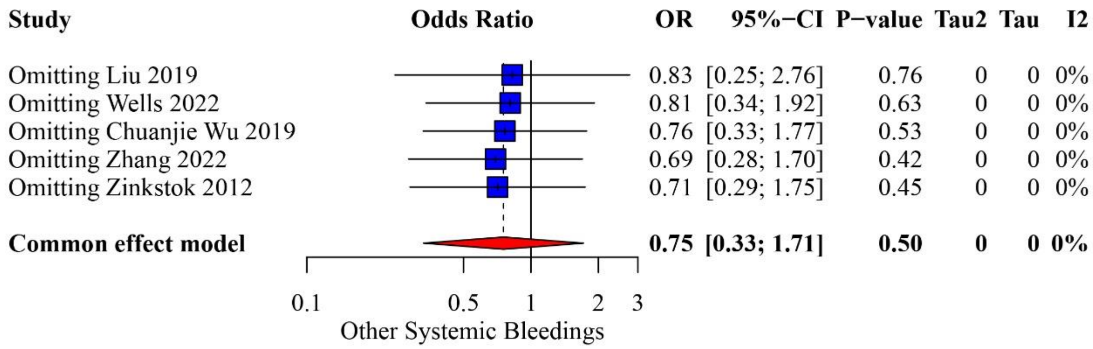

Figure S4: Forest plot for sensitivity analysis of other systemic bleedings outcome.

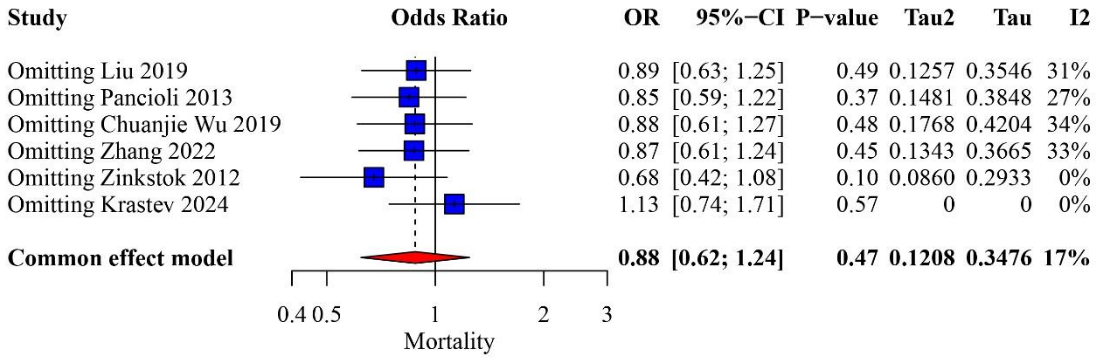

Figure S5: Forest plot for sensitivity analysis of mortality at 90 days.

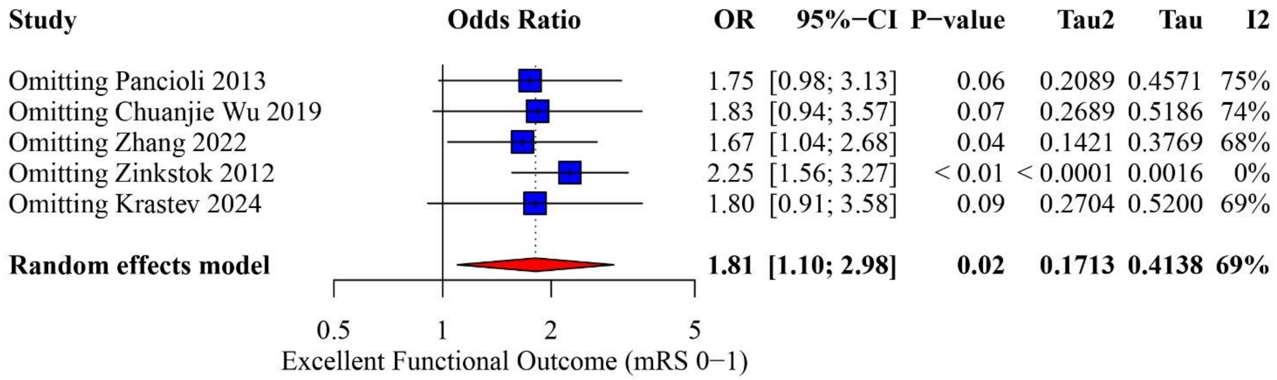

Figure S6: Forest plot for sensitivity analysis of excellent functional outcome (mRS 0-1).

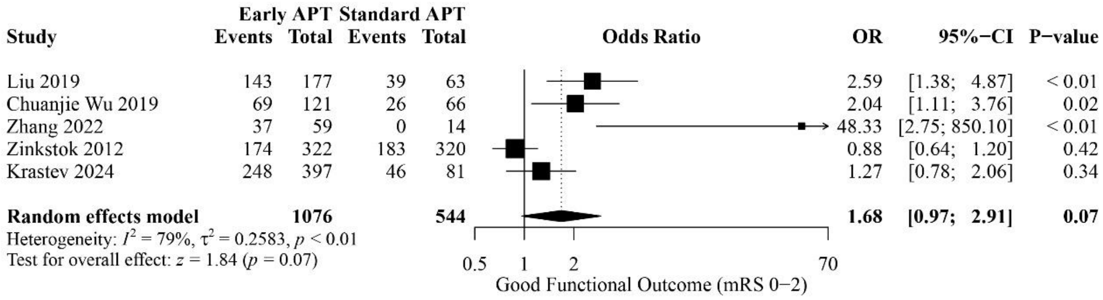

Figure S7: Forest plot for meta-analysis of good functional outcome (mRS 0-2).

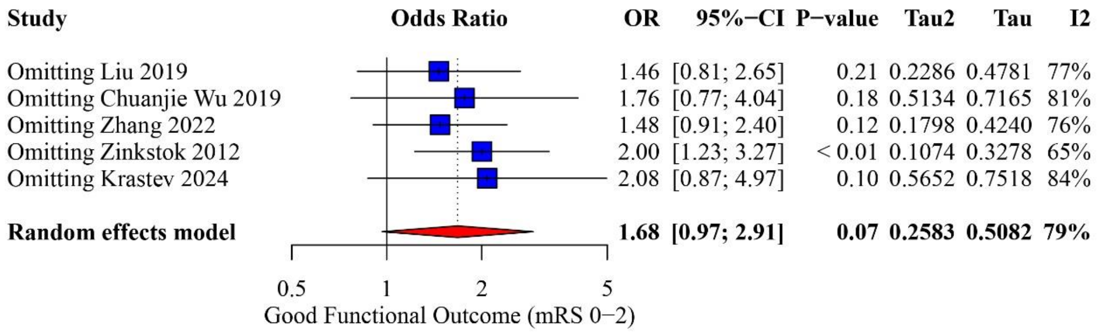

Figure S8: Forest plot for sensitivity analysis of good functional outcome (mRS 0-2).

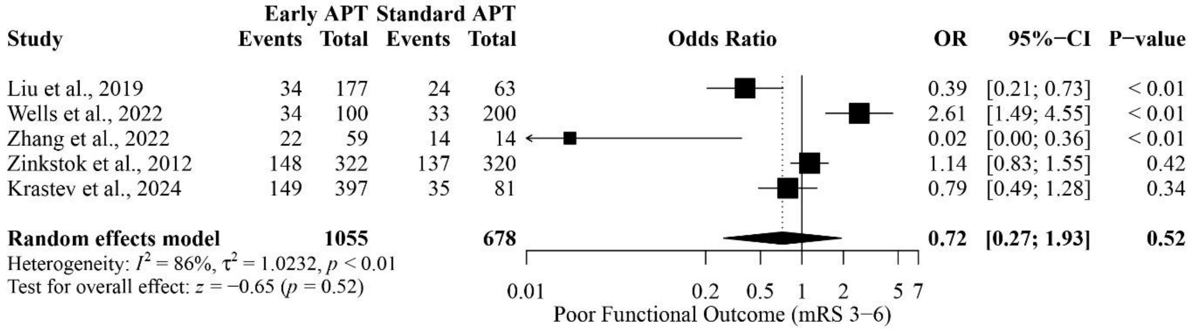

Figure S9: Forest plot for meta-analysis of poor functional outcome (mRS 3-6).

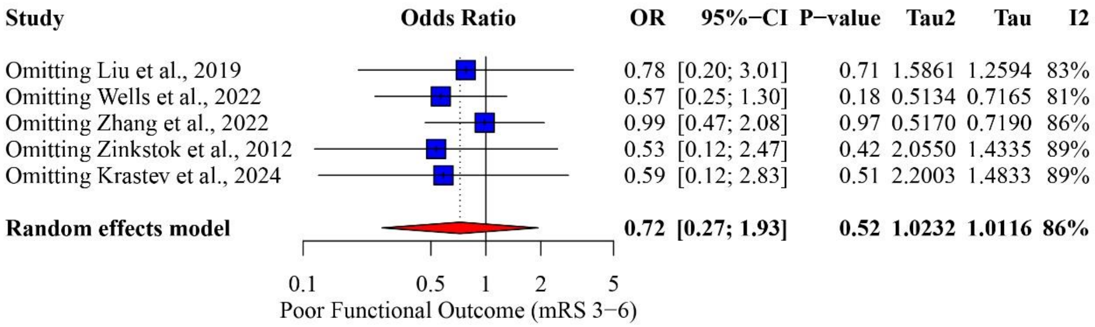

Figure S10: Forest plot for sensitivity analysis of poor functional outcome (mRS 3-6).

Study or Subgroup	Early APT Events	Early APT Total	Standard APT Events	Standard APT Total	Weight	Odds Ratio MH, Fixed, 95% CI
**Study design = Obsevational**						
Amaro 2013	1	52	0	25	4.4%	1.49 [0.06; 37.76]
Wells 2022	5	100	0	200	2.1%	23.09 [1.26; 421.95]
Chuanjie Wu 2019	2	121	1	66	8.6%	1.09 [0.10; 12.28]
Krastev 2024	0	407	0	82	5.6%	0.20 [0.00; 10.28]
**Total (95% CI)**		**680**		**373**	**20.7%**	**3.20 [0.87; 11.80]**
Heterogeneity: $Tau^2 = 0.9718$; $Chi^2 = 4.3$, df = 3 (P = 0.23); $I^2 = 30\%$						
Test for overall effect: Z = 1.75 (P = 0.08)						
**Study design = RCT**						
Liu 2019	1	177	1	63	9.9%	0.35 [0.02; 5.72]
Pancioli 2013	2	101	3	25	31.8%	0.15 [0.02; 0.94]
Zhang 2022	2	59	0	14	5.2%	1.26 [0.06; 27.72]
Zinkstok 2012	14	322	5	320	32.4%	2.86 [1.02; 8.05]
**Total (95% CI)**		**659**		**422**	**79.3%**	**1.36 [0.63; 2.91]**
Heterogeneity: $Tau^2 = 1.4942$; $Chi^2 = 8.38$, df = 3 (P = 0.04); $I^2 = 64\%$						
Test for overall effect: Z = 0.78 (P = 0.44)						
**Total (95% CI)**		**1339**		**795**	**100.0%**	**1.74 [0.91; 3.33]**
Heterogeneity: $Tau^2 = 1.1552$; $Chi^2 = 13.01$, df = 7 (P = 0.07); $I^2 = 46\%$						
Test for overall effect: Z = 1.66 (P = 0.10)						
Test for subgroup differences: $Chi^2 = 1.24$, df = 1 (P = 0.27)						

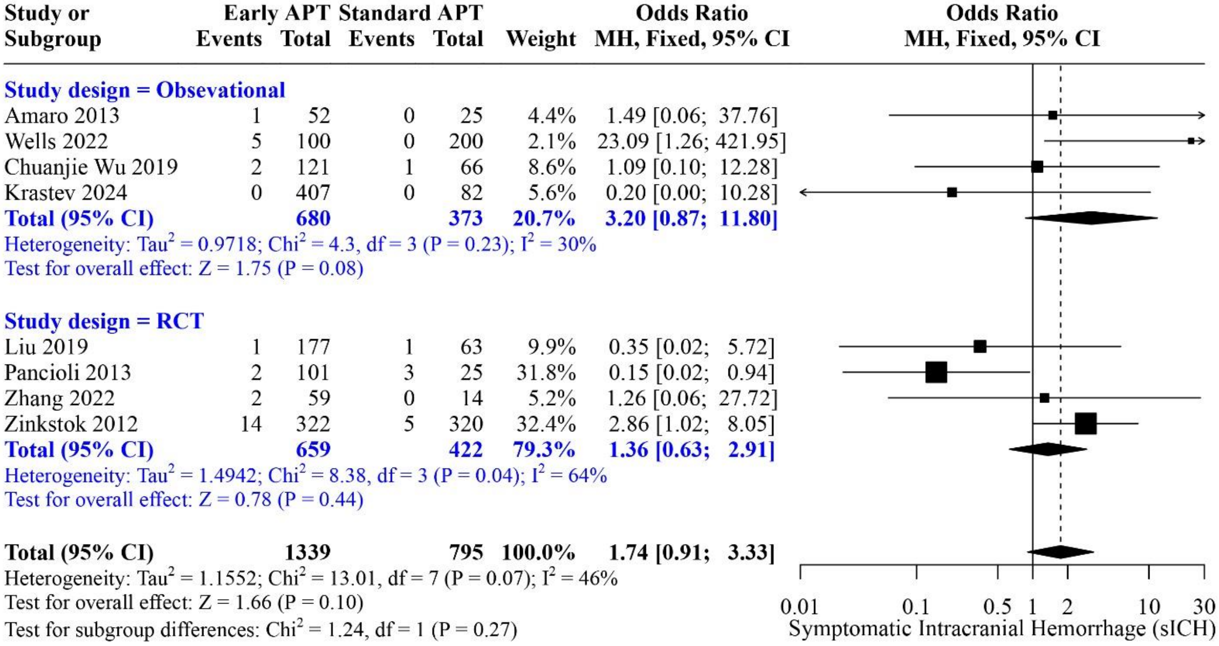

Figure S11: Subgroup analysis based on study design for sICH.

Study or Subgroup	Early APT Events	Early APT Total	Standard APT Events	Standard APT Total	Weight	Odds Ratio MH, Fixed, 95% CI
**Mean baseline NIHSS = (= 10)**						
Amaro 2013	1	52	0	25	4.4%	1.49 [0.06; 37.76]
Liu 2019	1	177	1	63	9.9%	0.35 [0.02; 5.72]
Chuanjie Wu 2019	2	121	1	66	8.6%	1.09 [0.10; 12.28]
Zhang 2022	2	59	0	14	5.2%	1.26 [0.06; 27.72]
Zinkstok 2012	14	322	5	320	32.4%	2.86 [1.02; 8.05]
Krastev 2024	0	407	0	82	5.6%	0.20 [0.00; 10.28]
**Total (95% CI)**		**1138**		**570**	**66.0%**	**1.81 [0.82; 4.02]**
Heterogeneity: $Tau^2 = 0.1403$; $Chi^2 = 3.5$, df = 5 (P = 0.62); $I^2 = 0\%$						
Test for overall effect: Z = 1.47 (P = 0.14)						
**Mean baseline NIHSS = (> 10)**						
Pancioli 2013	2	101	3	25	31.8%	0.15 [0.02; 0.94]
Wells 2022	5	100	0	200	2.1%	23.09 [1.26; 421.95]
**Total (95% CI)**		**201**		**225**	**34.0%**	**1.59 [0.51; 4.94]**
Heterogeneity: $Tau^2 = 11.2038$; $Chi^2 = 8.26$, df = 1 (P < 0.01); $I^2 = 88\%$						
Test for overall effect: Z = 0.80 (P = 0.42)						
**Total (95% CI)**		**1339**		**795**	**100.0%**	**1.74 [0.91; 3.33]**
Heterogeneity: $Tau^2 = 1.1552$; $Chi^2 = 13.01$, df = 7 (P = 0.07); $I^2 = 46\%$						
Test for overall effect: Z = 1.66 (P = 0.10)						
Test for subgroup differences: $Chi^2 = 0.03$, df = 1 (P = 0.85)						

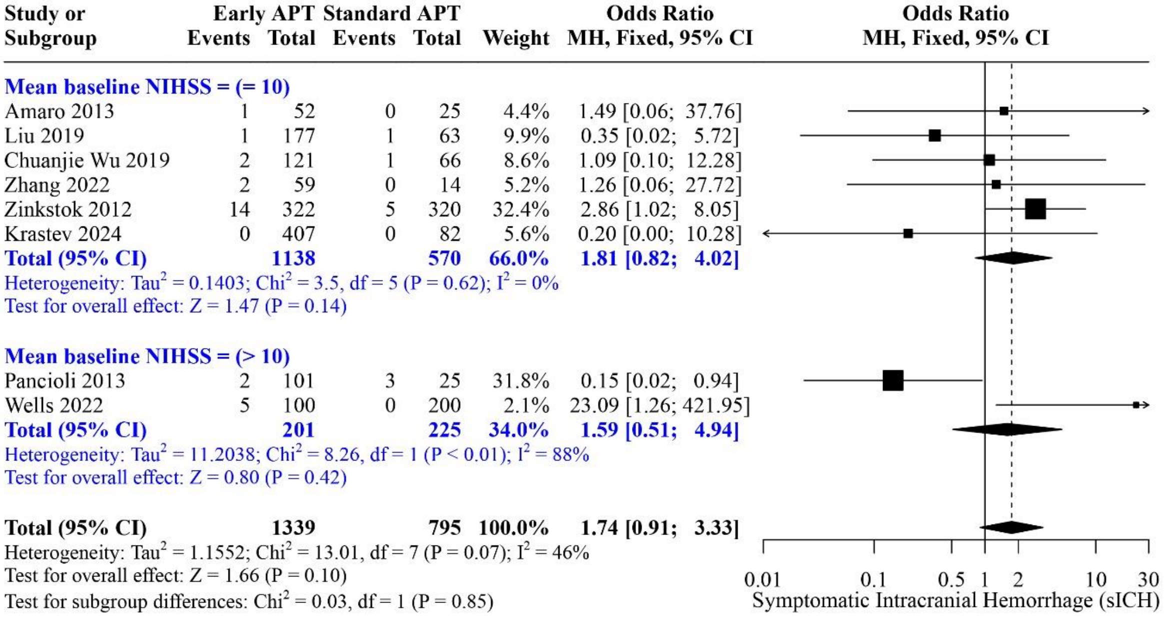

Figure S12: Subgroup analysis based on baseline NIHSS for sICH.

Study or Subgroup	Early APT Events	Early APT Total	Standard APT Events	Standard APT Total	Weight	Odds Ratio MH, Fixed, 95% CI
**Study design = Obsevational**						
Amaro 2013	6	52	1	25	6.0%	3.13 [0.36; 27.52]
Chuanjie Wu 2019	7	121	3	66	18.2%	1.29 [0.32; 5.16]
**Total (95% CI)**		**173**		**91**	**24.2%**	**1.74 [0.55; 5.52]**
Heterogeneity: $Tau^2 = 0$; $Chi^2 = 0.45$, df = 1 (P = 0.50); $I^2 = 0\%$						
Test for overall effect: Z = 0.95 (P = 0.34)						
**Study design = RCT**						
Liu 2019	8	177	4	63	28.1%	0.70 [0.20; 2.40]
Pancioli 2013	16	101	6	25	40.4%	0.60 [0.21; 1.72]
Zhang 2022	5	59	1	14	7.4%	1.20 [0.13; 11.20]
**Total (95% CI)**		**337**		**102**	**75.8%**	**0.69 [0.33; 1.47]**
Heterogeneity: $Tau^2 = 0$; $Chi^2 = 0.31$, df = 2 (P = 0.86); $I^2 = 0\%$						
Test for overall effect: Z = −0.95 (P = 0.34)						
**Total (95% CI)**		**510**		**193**	**100.0%**	**0.95 [0.51; 1.77]**
Heterogeneity: $Tau^2 = 0$; $Chi^2 = 2.33$, df = 4 (P = 0.68); $I^2 = 0\%$						
Test for overall effect: Z = −0.17 (P = 0.86)						
Test for subgroup differences: $Chi^2 = 1.73$, df = 1 (P = 0.19)						

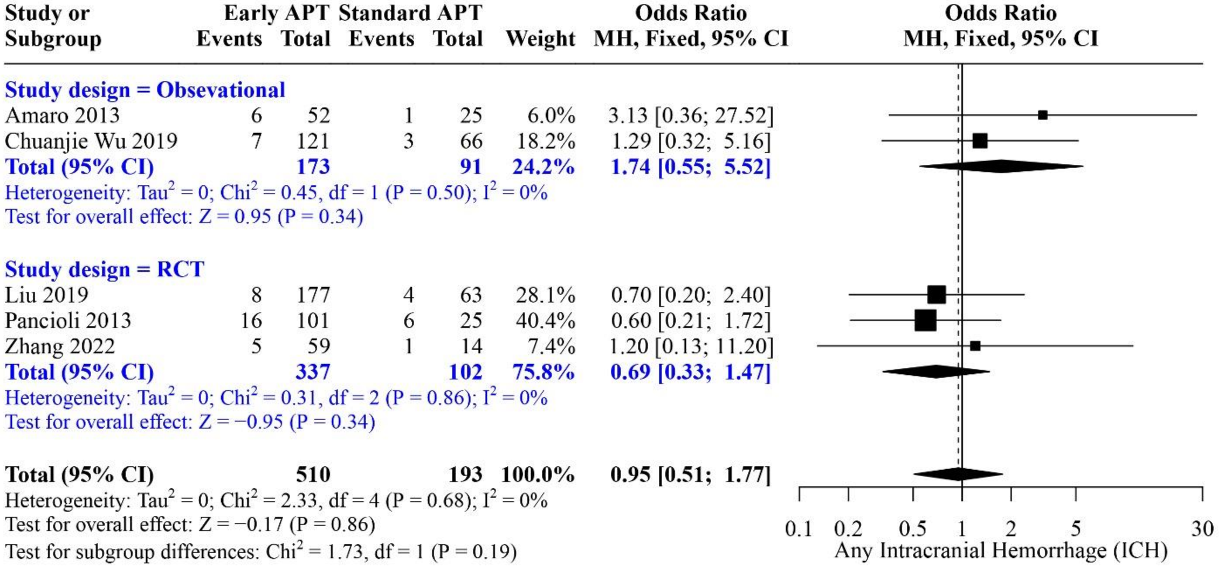

Figure S13: Subgroup analysis based on study design for any ICH.

Study or Subgroup	Early APT Events	Early APT Total	Standard APT Events	Standard APT Total	Weight	Odds Ratio MH, Fixed, 95% CI
**Mean baseline NIHSS = (= 10)**						
Amaro 2013	6	52	1	25	6.0%	3.13 [0.36; 27.52]
Liu 2019	8	177	4	63	28.1%	0.70 [0.20; 2.40]
Chuanjie Wu 2019	7	121	3	66	18.2%	1.29 [0.32; 5.16]
Zhang 2022	5	59	1	14	7.4%	1.20 [0.13; 11.20]
**Total (95% CI)**		**409**		**168**	**59.6%**	**1.18 [0.54; 2.59]**
Heterogeneity: $Tau^2 = 0$; $Chi^2 = 1.46$, df = 3 (P = 0.69); $I^2 = 0\%$						
Test for overall effect: Z = 0.42 (P = 0.67)						
**Mean baseline NIHSS = (> 10)**						
Pancioli 2013	16	101	6	25	40.4%	0.60 [0.21; 1.72]
**Total (95% CI)**		**510**		**193**	**100.0%**	**0.95 [0.51; 1.77]**
Heterogeneity: $Tau^2 = 0$; $Chi^2 = 2.33$, df = 4 (P = 0.68); $I^2 = 0\%$						
Test for overall effect: Z = −0.17 (P = 0.86)						
Test for subgroup differences: $Chi^2 = 1.04$, df = 1 (P = 0.31)						

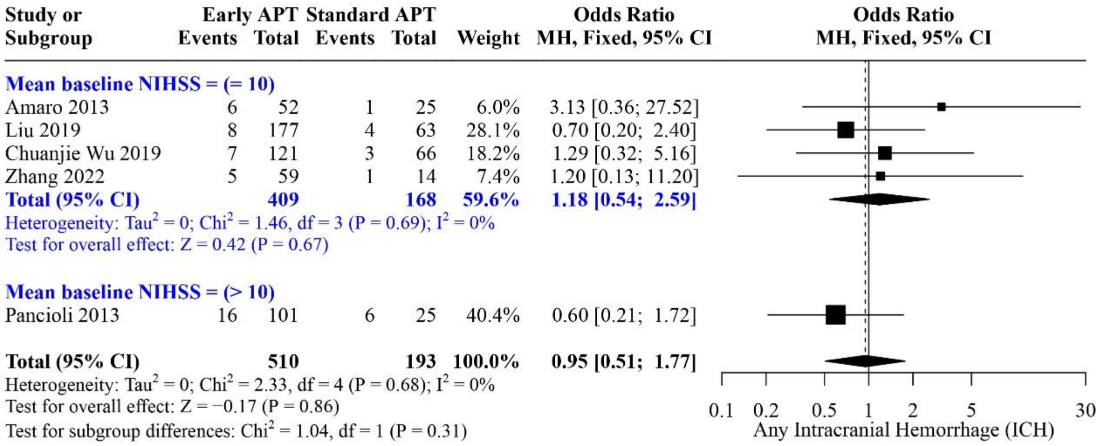

Figure S14: Subgroup analysis based on baseline NIHSS for any ICH.

Study or Subgroup	Early APT Events	Early APT Total	Standard APT Events	Standard APT Total	Weight	Odds Ratio MH, Fixed, 95% CI
**Study design = RCT**						
Liu 2019	10	177	5	63	54.6%	0.69 [0.23; 2.12]
Zhang 2022	5	59	1	14	11.6%	1.20 [0.13; 11.20]
Zinkstok 2012	2	322	2	320	15.7%	0.99 [0.14; 7.10]
**Total (95% CI)**		**558**		**397**	**81.9%**	**0.82 [0.34; 2.00]**
Heterogeneity: $Tau^2 = 0$; $Chi^2 = 0.24$, df = 2 (P = 0.89); $I^2 = 0\%$						
Test for overall effect: Z = −0.43 (P = 0.67)						
**Study design = Obsevational**						
Wells 2022	0	100	2	200	13.1%	0.40 [0.02; 8.31]
Chuanjie Wu 2019	0	121	0	66	5.0%	0.55 [0.01; 27.90]
**Total (95% CI)**		**221**		**266**	**18.1%**	**0.44 [0.04; 4.81]**
Heterogeneity: $Tau^2 = 0$; $Chi^2 = 0.02$, df = 1 (P = 0.90); $I^2 = 0\%$						
Test for overall effect: Z = −0.68 (P = 0.50)						
**Total (95% CI)**		**779**		**663**	**100.0%**	**0.75 [0.33; 1.71]**

Heterogeneity: $Tau^2 = 0$; $Chi^2 = 0.46$, df = 4 (P = 0.98); $I^2 = 0\%$
Test for overall effect: Z = −0.67 (P = 0.50)
Test for subgroup differences: $Chi^2 = 0.24$, df = 1 (P = 0.63)

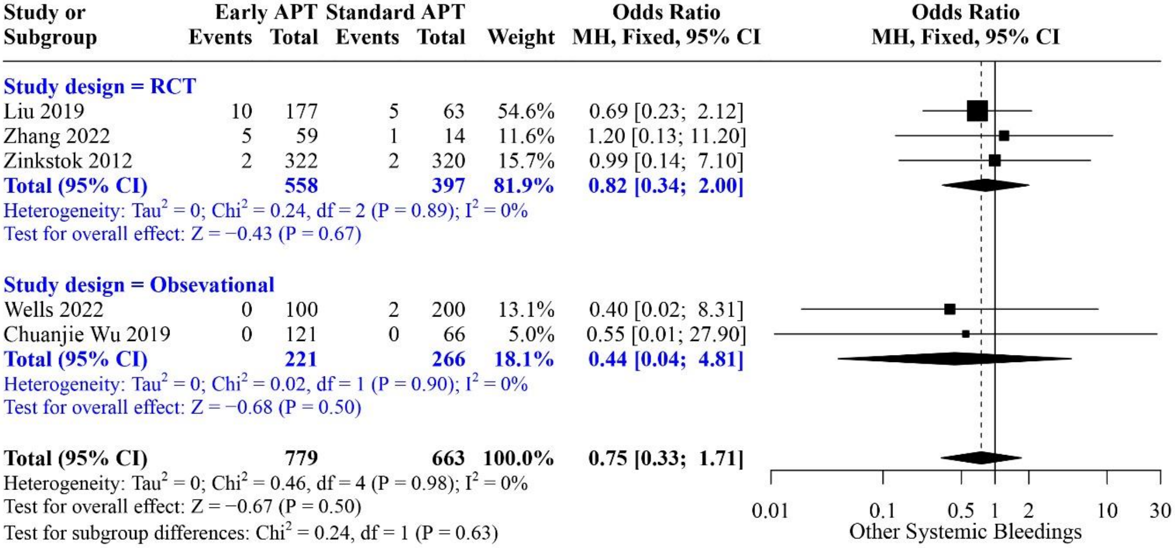

Figure S15: Subgroup analysis based on study design for other systemic bleedings.

Study or Subgroup	Early APT Events	Early APT Total	Standard APT Events	Standard APT Total	Weight	Odds Ratio MH, Fixed, 95% CI
**Mean baseline NIHSS = (= 10)**						
Liu 2019	10	177	5	63	54.6%	0.69 [0.23; 2.12]
Chuanjie Wu 2019	0	121	0	66	5.0%	0.55 [0.01; 27.90]
Zhang 2022	5	59	1	14	11.6%	1.20 [0.13; 11.20]
Zinkstok 2012	2	322	2	320	15.7%	0.99 [0.14; 7.10]
**Total (95% CI)**		**679**		**463**	**86.9%**	**0.81 [0.34; 1.92]**
Heterogeneity: $Tau^2 = 0$; $Chi^2 = 0.27$, df = 3 (P = 0.97); $I^2 = 0\%$						
Test for overall effect: Z = −0.48 (P = 0.63)						
**Mean baseline NIHSS = (> 10)**						
Wells 2022	0	100	2	200	13.1%	0.40 [0.02; 8.31]
**Total (95% CI)**		**779**		**663**	**100.0%**	**0.75 [0.33; 1.71]**
Heterogeneity: $Tau^2 = 0$; $Chi^2 = 0.46$, df = 4 (P = 0.98); $I^2 = 0\%$						
Test for overall effect: Z = −0.67 (P = 0.50)						
Test for subgroup differences: $Chi^2 = 0.20$, df = 1 (P = 0.66)						

Odds Ratio
MH, Fixed, 95% CI

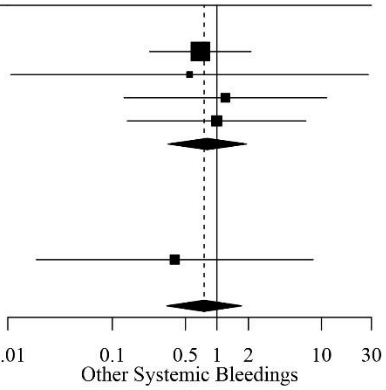

Figure S16: Subgroup analysis based on baseline NIHSS for other systemic bleeding outcome.

Study or Subgroup	Early APT Events	Early APT Total	Standard APT Events	Standard APT Total	Weight	Odds Ratio MH, Fixed, 95% CI
**Study design = RCT**						
Liu 2019	0	177	0	63	1.1%	0.36 [0.01; 18.22]
Pancioli 2013	20	101	4	25	7.6%	1.30 [0.40; 4.20]
Zhang 2022	5	59	1	14	2.2%	1.20 [0.13; 11.20]
Zinkstok 2012	36	322	31	320	41.0%	1.17 [0.71; 1.95]
**Total (95% CI)**		**659**		**422**	**51.9%**	**1.18 [0.75; 1.85]**
Heterogeneity: $Tau^2 = 0$; $Chi^2 = 0.38$, df = 3 (P = 0.94); $I^2 = 0\%$						
Test for overall effect: Z = 0.70 (P = 0.48)						
**Study design = Obsevational**						
Chuanjie Wu 2019	10	121	6	66	10.6%	0.90 [0.31; 2.60]
Krastev 2024	44	407	17	82	37.5%	0.46 [0.25; 0.86]
**Total (95% CI)**		**528**		**148**	**48.1%**	**0.56 [0.33; 0.96]**
Heterogeneity: $Tau^2 = 0.0249$; $Chi^2 = 1.13$, df = 1 (P = 0.29); $I^2 = 11\%$						
Test for overall effect: Z = −2.12 (P = 0.03)						
**Total (95% CI)**		**1187**		**570**	**100.0%**	**0.88 [0.62; 1.24]**
Heterogeneity: $Tau^2 = 0.1208$; $Chi^2 = 6.03$, df = 5 (P = 0.30); $I^2 = 17\%$						
Test for overall effect: Z = −0.73 (P = 0.47)						
Test for subgroup differences: $Chi^2 = 4.30$, df = 1 (P = 0.04)						

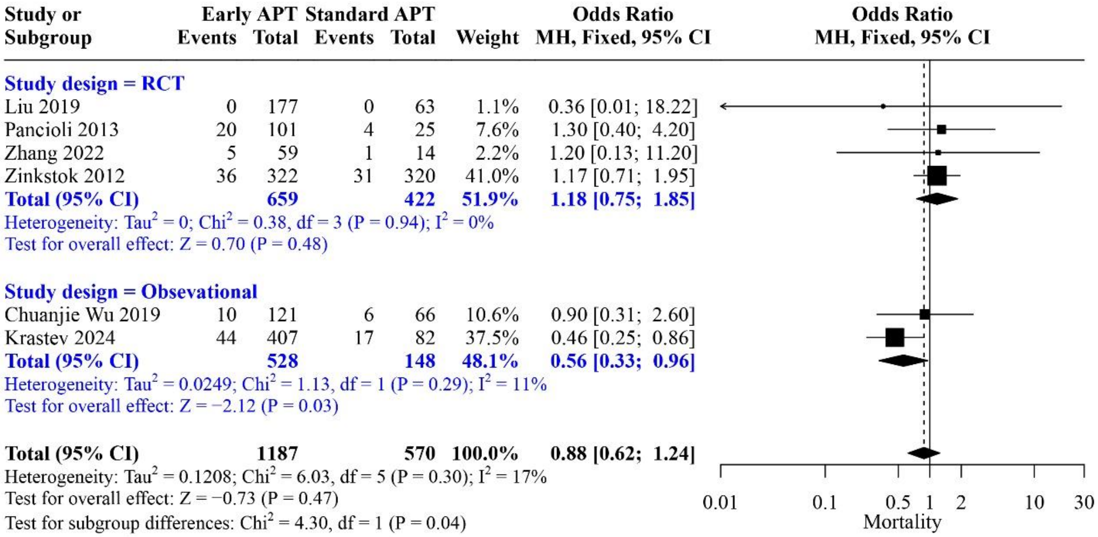

Figure S17: Subgroup analysis based on study design for mortality.

Study or Subgroup	Early APT Events	Early APT Total	Standard APT Events	Standard APT Total	Weight	Odds Ratio MH, Fixed, 95% CI
**Mean baseline NIHSS = (= 10)**						
Liu 2019	0	177	0	63	1.1%	0.36 [0.01; 18.22]
Chuanjie Wu 2019	10	121	6	66	10.6%	0.90 [0.31; 2.60]
Zhang 2022	5	59	1	14	2.2%	1.20 [0.13; 11.20]
Zinkstok 2012	36	322	31	320	41.0%	1.17 [0.71; 1.95]
Krastev 2024	44	407	17	82	37.5%	0.46 [0.25; 0.86]
**Total (95% CI)**		**1086**		**545**	**92.4%**	**0.85 [0.59; 1.22]**
Heterogeneity: $Tau^2 = 0.1481$; $Chi^2 = 5.5$, df = 4 (P = 0.24); $I^2 = 27\%$						
Test for overall effect: Z = −0.91 (P = 0.37)						
**Mean baseline NIHSS = (> 10)**						
Pancioli 2013	20	101	4	25	7.6%	1.30 [0.40; 4.20]
**Total (95% CI)**		**1187**		**570**	**100.0%**	**0.88 [0.62; 1.24]**
Heterogeneity: $Tau^2 = 0.1208$; $Chi^2 = 6.03$, df = 5 (P = 0.30); $I^2 = 17\%$						
Test for overall effect: Z = −0.73 (P = 0.47)						
Test for subgroup differences: $Chi^2 = 0.46$, df = 1 (P = 0.50)						

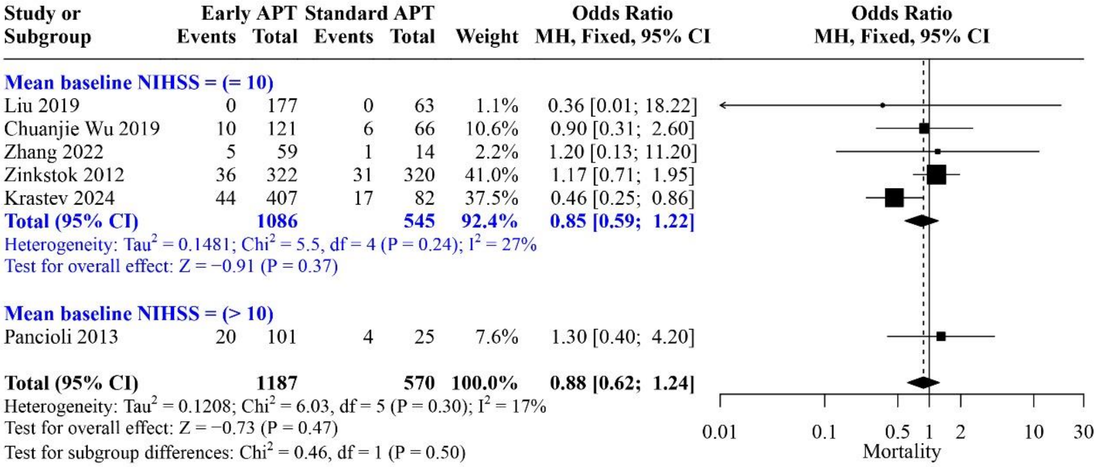

Figure S18: Subgroup analysis based on baseline NIHSS for mortality.

Study or Subgroup	Early APT Events	Early APT Total	Standard APT Events	Standard APT Total	Weight	Odds Ratio MH, Random, 95% CI
**Study design = RCT**						
Pancioli 2013	44	101	6	25	15.1%	2.44 [0.90; 6.64]
Zhang 2022	26	59	0	14	2.8%	22.94 [1.31; 402.53]
Zinkstok 2012	100	322	100	320	32.4%	0.99 [0.71; 1.38]
**Total (95% CI)**		**482**		**359**	**50.3%**	**2.17 [0.61; 7.70]**
Heterogeneity: $Tau^2 = 0.8412$; $Chi^2 = 7.13$, df = 2 (P = 0.03); $I^2 = 72\%$						
Test for overall effect: Z = 1.20 (P = 0.23)						
**Study design = Obsevational**						
Chuanjie Wu 2019	46	121	15	66	22.2%	2.09 [1.05; 4.13]
Krastev 2024	211	397	28	81	27.5%	2.15 [1.30; 3.54]
**Total (95% CI)**		**518**		**147**	**49.7%**	**2.13 [1.42; 3.18]**
Heterogeneity: $Tau^2 = 0$; $Chi^2 = 0$, df = 1 (P = 0.95); $I^2 = 0\%$						
Test for overall effect: Z = 3.67 (P < 0.01)						
**Total (95% CI)**		**1000**		**506**	**100.0%**	**1.81 [1.10; 2.98]**
Heterogeneity: $Tau^2 = 0.1713$; $Chi^2 = 13.07$, df = 4 (P = 0.01); $I^2 = 69\%$						
Test for overall effect: Z = 2.33 (P = 0.02)						
Test for subgroup differences: $Chi^2 = 0.00$, df = 1 (P = 0.98)						

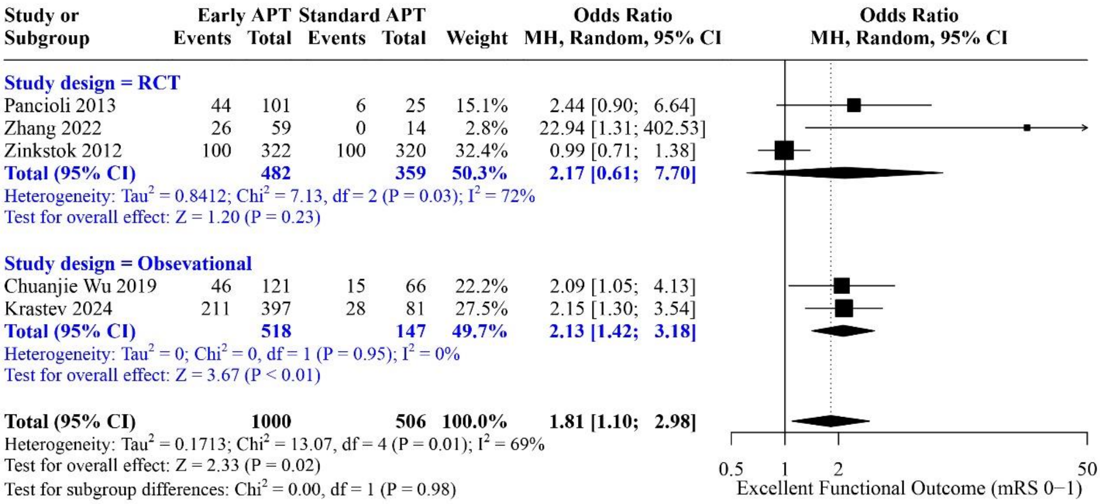

Figure S19: Subgroup analysis based on study design for mRS 0-1 outcome.

Study or Subgroup	Early APT Events	Early APT Total	Standard APT Events	Standard APT Total	Weight	Odds Ratio MH, Random, 95% CI
**Mean baseline NIHSS = (> 10)**						
Pancioli 2013	44	101	6	25	15.1%	2.44 [0.90; 6.64]
**Mean baseline NIHSS = (= 10)**						
Chuanjie Wu 2019	46	121	15	66	22.2%	2.09 [1.05; 4.13]
Zhang 2022	26	59	0	14	2.8%	22.94 [1.31; 402.53]
Zinkstok 2012	100	322	100	320	32.4%	0.99 [0.71; 1.38]
Krastev 2024	211	397	28	81	27.5%	2.15 [1.30; 3.54]
**Total (95% CI)**		**899**		**481**	**84.9%**	**1.75 [0.98; 3.13]**
Heterogeneity: $Tau^2 = 0.2089$; $Chi^2 = 11.9$, df = 3 ($P < 0.01$); $I^2 = 75\%$						
Test for overall effect: Z = 1.88 (P = 0.06)						
**Total (95% CI)**		**1000**		**506**	**100.0%**	**1.81 [1.10; 2.98]**
Heterogeneity: $Tau^2 = 0.1713$; $Chi^2 = 13.07$, df = 4 ($P = 0.01$); $I^2 = 69\%$						
Test for overall effect: Z = 2.33 (P = 0.02)						
Test for subgroup differences: $Chi^2 = 0.32$, df = 1 ($P = 0.57$)						

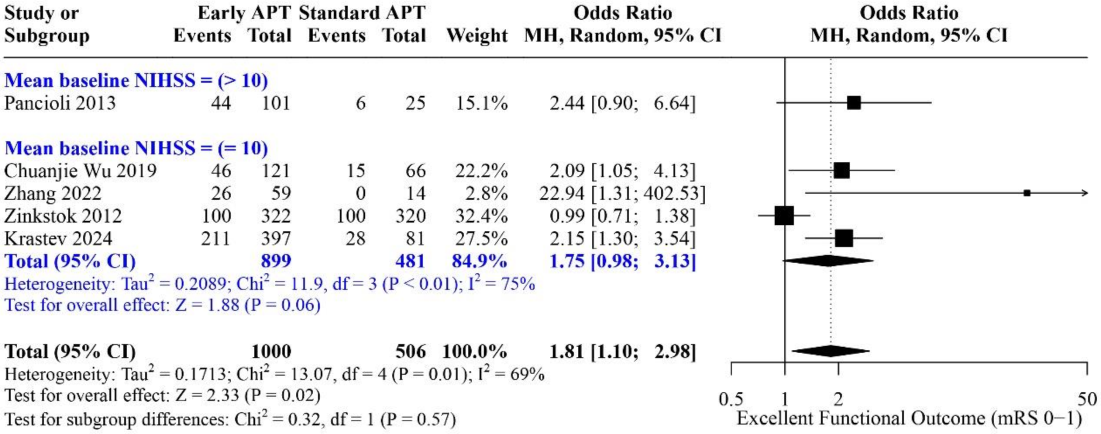

Figure S20: Subgroup analysis based on baseline NIHSS for mRS 0-1 outcome.

Study or Subgroup	Early APT Events	Early APT Total	Standard APT Events	Standard APT Total	Weight	Odds Ratio MH, Random, 95% CI
**Study design = RCT**						
Liu et al., 2019	143	177	39	63	21.8%	2.59 [1.38; 4.87]
Zhang et al., 2022	37	59	0	14	3.3%	48.33 [2.75; 850.10]
Zinkstok et al., 2012	174	322	183	320	27.9%	0.88 [0.64; 1.20]
**Total (95% CI)**		**558**		**397**	**53.0%**	**3.09 [0.47; 20.14]**
Heterogeneity: $Tau^2 = 2.2499$; $Chi^2 = 15.69$, df = 2 ($P < 0.01$); $I^2 = 87\%$						
Test for overall effect: $Z = 1.18$ ($P = 0.24$)						
**Study design = Obsevational**						
Wu et al., 2019	69	121	26	66	22.2%	2.04 [1.11; 3.76]
Krastev et al., 2024	248	397	46	81	24.8%	1.27 [0.78; 2.06]
**Total (95% CI)**		**518**		**147**	**47.0%**	**1.55 [0.98; 2.46]**
Heterogeneity: $Tau^2 = 0.0349$; $Chi^2 = 1.44$, df = 1 ($P = 0.23$); $I^2 = 31\%$						
Test for overall effect: $Z = 1.85$ ($P = 0.06$)						
**Total (95% CI)**		**1076**		**544**	**100.0%**	**1.68 [0.97; 2.91]**
Heterogeneity: $Tau^2 = 0.2583$; $Chi^2 = 18.70$, df = 4 ($P < 0.01$); $I^2 = 79\%$						
Test for overall effect: $Z = 1.84$ ($P = 0.07$)						
Test for subgroup differences: $Chi^2 = 0.49$, df = 1 ($P = 0.48$)						

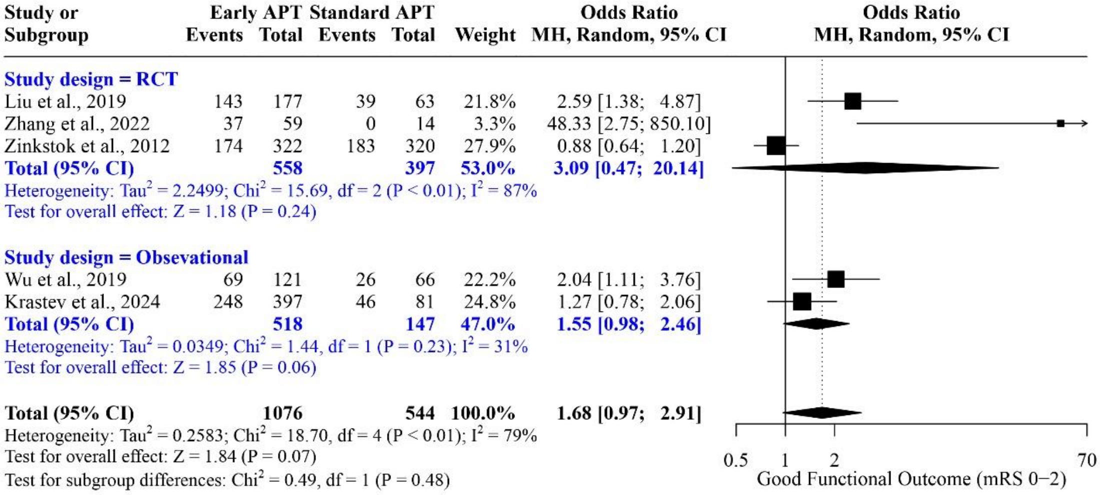

Figure S21: Subgroup analysis based on study design for mRS 0-2 outcome.

Study or Subgroup	Early APT Events	Early APT Total	Standard APT Events	Standard APT Total	Weight	Odds Ratio MH, Random, 95% CI
**Study design = RCT**						
Liu et al., 2019	34	177	24	63	22.3%	0.39 [0.21; 0.73]
Zhang et al., 2022	22	59	14	14	7.9%	0.02 [0.00; 0.36]
Zinkstok et al., 2012	148	322	137	320	23.9%	1.14 [0.83; 1.55]
**Total (95% CI)**		**558**		**397**	**54.1%**	**0.32 [0.05; 2.11]**
Heterogeneity: $Tau^2 = 2.2499$; $Chi^2 = 15.69$, df = 2 ($P < 0.01$); $I^2 = 87\%$						
Test for overall effect: $Z = -1.18$ ($P = 0.24$)						
**Study design = Obsevational**						
Wells et al., 2022	34	100	33	200	22.7%	2.61 [1.49; 4.55]
Krastev et al., 2024	149	397	35	81	23.1%	0.79 [0.49; 1.28]
**Total (95% CI)**		**497**		**281**	**45.9%**	**1.42 [0.44; 4.59]**
Heterogeneity: $Tau^2 = 0.6423$; $Chi^2 = 10.05$, df = 1 ($P < 0.01$); $I^2 = 90\%$						
Test for overall effect: $Z = 0.59$ ($P = 0.55$)						
**Total (95% CI)**		**1055**		**678**	**100.0%**	**0.72 [0.27; 1.93]**
Heterogeneity: $Tau^2 = 1.0232$; $Chi^2 = 28.61$, df = 4 ($P < 0.01$); $I^2 = 86\%$						
Test for overall effect: $Z = -0.65$ ($P = 0.52$)						
Test for subgroup differences: $Chi^2 = 1.72$, df = 1 ($P = 0.19$)						

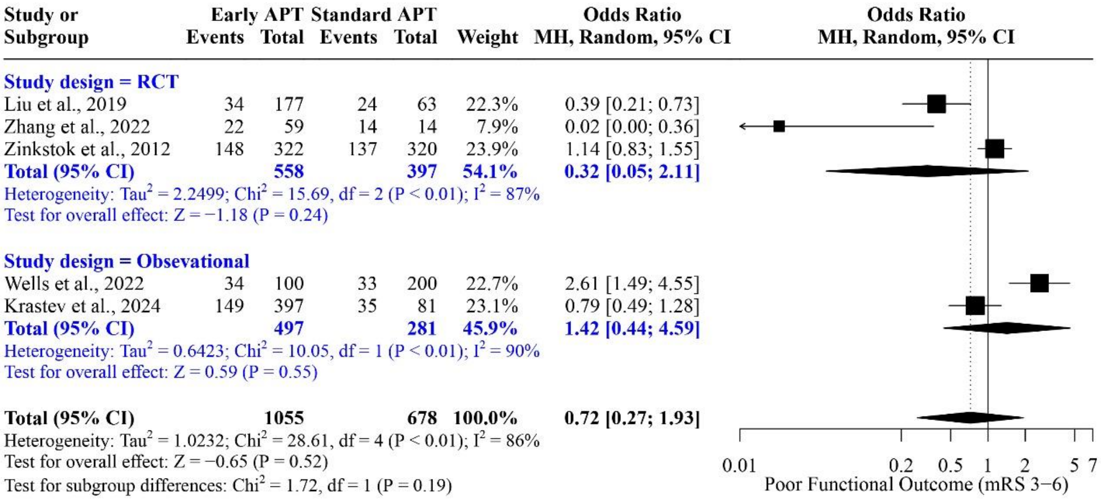

Figure S22: Subgroup analysis based on study design for mRS 3-6 outcome.

Study or Subgroup	Early APT Events	Early APT Total	Standard APT Events	Standard APT Total	Weight	Odds Ratio MH, Random, 95% CI
**Mean baseline NIHSS = (= 10)**						
Liu 2019	34	177	24	63	22.3%	0.39 [0.21; 0.73]
Zhang 2022	22	59	14	14	7.9%	0.02 [0.00; 0.36]
Zinkstok 2012	148	322	137	320	23.9%	1.14 [0.83; 1.55]
Krastev 2024	149	397	35	81	23.1%	0.79 [0.49; 1.28]
**Total (95% CI)**		**955**		**478**	**77.3%**	**0.57 [0.25; 1.30]**
Heterogeneity: $Tau^2 = 0.5134$; $Chi^2 = 15.85$, df = 3 ($P < 0.01$); $I^2 = 81\%$						
Test for overall effect: $Z = -1.34$ ($P = 0.18$)						
**Mean baseline NIHSS = (> 10)**						
Wells 2022	34	100	33	200	22.7%	2.61 [1.49; 4.55]
**Total (95% CI)**		**1055**		**678**	**100.0%**	**0.72 [0.27; 1.93]**
Heterogeneity: $Tau^2 = 1.0232$; $Chi^2 = 28.61$, df = 4 ($P < 0.01$); $I^2 = 86\%$						
Test for overall effect: $Z = -0.65$ ($P = 0.52$)						
Test for subgroup differences: $Chi^2 = 8.97$, df = 1 ($P < 0.01$)						

Odds Ratio
MH, Random, 95% CI

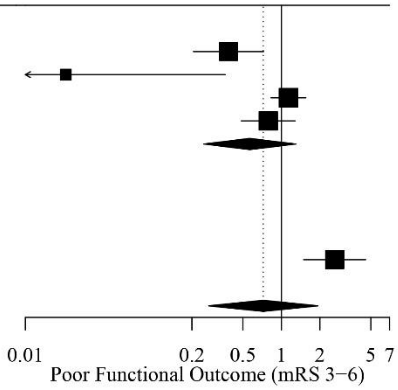

Figure S23: Subgroup analysis based on baseline NIHSS for mRS 3-6 outcome.
